# Characteristics of prescription in 29 Level 3 Neonatal Wards over a 2-year period (2017-2018). An inventory for future research

**DOI:** 10.1371/journal.pone.0222667

**Published:** 2019-09-19

**Authors:** Béatrice Gouyon, Séverine Martin-Mons, Silvia Iacobelli, Hasinirina Razafimahefa, Elsa Kermorvant-Duchemin, Roselyne Brat, Laurence Caeymaex, Yvan Couringa, Ceneric Alexandre, Catherine Lafon, Duksha Ramful, Francesco Bonsante, Guillaume Binson, Florence Flamein, Amélie Moussy-Durandy, Massimo Di Maio, Gaël Mazeiras, Olivier Girard, Cécile Desbruyeres, Julien Mourdie, Guillaume Escourrou, Olivier Flechelles, Soumeth Abasse, Jean-Marc Rosenthal, Anne-Sophie Pages, Marine Dorsi, Léila Karaoui, Abdellah ElGellab, Florence Le Bail Dantec, Mohamed-Amine Yangui, Karine Norbert, Yaovi Kugbe, Simon Lorrain, Anaelle Pignolet, Elodie Marie Garnier, Alexandre Lapillonne, Delphine Mitanchez, Evelyne Jacqz-Aigrain, Jean-Bernard Gouyon

**Affiliations:** 1 Centre d’Etudes Périnatales de l’Océan Indien (EA 7388), Centre Hospitalier Universitaire de La Réunion – Site Sud, Saint Pierre, Réunion, France; 2 Centre Hospitalier Sud Francilien, Corbeil-Essonnes, France; 3 Hôpital Necker-Enfants Malades, Paris, France; 4 Centre Hospitalier Régional d’Orléans, Orléans, France; 5 Centre Hospitalier Intercommunal de Créteil, Créteil, France; 6 Centre Hospitalier Andrée-Rosemon, Guyane Française, France; 7 Centre Hospitalier Universitaire de Caen, Caen, France; 8 Centre Hospitalier d’Arras, Arras, France; 9 Centre Hospitalier Universitaire de La Réunion – Site Nord, Saint Denis, Réunion, France; 10 Centre Hospitalier Universitaire de Poitiers, Poitiers, France; 11 Centre Hospitalier Universitaire de Lille, Lille, France; 12 Centre Hospitalier Intercommunal Poissy/Saint Germain en Laye, Poissy, France; 13 Centre Hospitalier Universitaire de Nîmes, Nîmes, France; 14 Centre Hospitalier de la Côte Basque, Bayonne, France; 15 Centre Hospitalier de Saint Denis, Saint Denis, France; 16 Centre Hospitalier Métropole Savoie, Chambéry, France; 17 Hôpital Jacques Monod – Groupe Hospitalier du Havre, Montivilliers, France; 18 Centre Hospitalier Intercommunal André Grégoire, Montreuil, France; 19 Centre Hospitalier Universitaire de Fort-de-France, Fort de France, Martinique, France; 20 Centre Hospitalier de Mayotte, Mayotte, France; 21 Centre Hospitalier Universitaire de Pointe-à-Pitre, Guadeloupe, France; 22 Centre Hospitalier Public du Cotentin, Cherbourg-en-Cotentin, France; 23 Centre Hospitalier Territorial Gaston-Bourret, Dumbéa, Nouvelle Calédonie, France; 24 Grand Hôpital de l’Est Francilien, Meaux, France; 25 Centre Hospitalier de Lens, Lens, France; 26 Centre Hospitalier de Saint Brieuc, Saint Brieuc, France; 27 Hôpital René Dubos, Pontoise, France; 28 Centre Hospitalier de Pau, Pau, France; 29 Centre Hospitalier de l’Ouest Guyanais – Franck Joly, Saint Laurent du Maroni, Guyane Française, France; 30 Hôpital Armand-Trousseau, Paris, France; 31 Hôpital Robert Debré, Paris, France; Centre Hospitalier Universitaire Vaudois, FRANCE

## Abstract

**Objectives:**

The primary objective of this study is to determine the current level of patient medication exposure in Level 3 Neonatal Wards (L3NW). The secondary objective is to evaluate in the first month of life the rate of medication prescription not cited in the Summary of Product Characteristics (SmPC). A database containing all the medication prescriptions is collected as part of a prescription benchmarking program in the L3NW.

**Material and methods:**

The research is a two-year observational cohort study (2017–2018) with retrospective analysis of medications prescribed in 29 French L3NW. Seventeen L3NW are present since the beginning of the study and 12 have been progressively included. All neonatal units used the same computerized system of prescription, and all prescription data were completely de-identified within each hospital before being stored in a common data warehouse.

**Results:**

The study population includes 27,382 newborns. Two hundred and sixty-one different medications (International Nonproprietary Names, INN) were prescribed. Twelve INN (including paracetamol) were prescribed for at least 10% of patients, 55 for less than 10% but at least 1% and 194 to less than 1%. The lowest gestational ages (GA) were exposed to the greatest number of medications (18.0 below 28 weeks of gestation (WG) to 4.1 above 36 WG) (p<0.0001). In addition, 69.2% of the 351 different combinations of an medication INN and a route of administration have no indication for the first month of life according to the French SmPC. Ninety-five percent of premature infants with GA less than 32 weeks received at least one medication not cited in SmPC.

**Conclusion:**

Neonates remain therapeutic orphans. The consequences of polypharmacy in L3NW should be quickly assessed, especially in the most immature infants.

## Introduction

Neonates in neonatal intensive care units (NICU) are exposed to the highest rates of unlicensed and off-label (UOL) medication prescription as compared to all hospitalized patients [1–4]. These babies have also the greatest risk of medication errors and adverse medication events [5]. The scarcity of high quality randomized controlled trials is considered the main contributor to this situation. Post-marketing medication surveillance should be particularly valuable in neonatal wards where information about medication efficacy and safety is so limited and insufficient [3, 4, 6].

A comprehensive and extensive systematic review of observational studies until the year 2016 [3] identified neonatal studies encompassing all medication classes. The most commonly reported medication studies were anti-infectives for systemic use followed by medications for cardiovascular system, nervous system and respiratory system. A more recent systematic review added 30 papers [4] and showed the diversity in the countries at the origin of the publications. The retained studies were either general or more specific of some International Nonproprietary Names (INN) and medication categories, such as anti-infectives, inotropics, surfactant, nitric oxide, narcotics-sedatives, caffeine, histamine-2 receptor antagonists and proton pump inhibitors. This list can be completed by recent papers focused on corticosteroids [7], antihypertensives [8] and paralyzers [9].

This study is the first part of a benchmarking program on prescription in neonatology, based on a single database of all electronic prescriptions performed over a 2-year period in 29 French Level 3 neonatal wards (i.e. with neonatal intensive care, intermediate care and neonatal medicine). The primary objective of this study is to determine the current level of patient medication exposure in the Level 3 Neonatal Wards (L3NW) from admission to discharge. The secondary objective is to evaluate the rate of exposure to medications not cited in the Summary of Product Characteristics (SmPC) for an administration in the first month of life.

## Material and methods

### The Level 3 Neonatal Wards of the study

From January 1, 2017 to December 31, 2018, twenty-nine L3NW engaged in a benchmarking program of neonatal medication prescribing practices (B-PEN program). Seventeen L3NW have been involved since the beginning, while another 12 have progressively joined the program. Prescription data were collected in the L3NW through a computerized order-entry system (CPOE) associated with a Clinical Decision Support System (CDS) (Logipren comp.). The study is limited to two years of data collection (2017–2018) to limit heterogeneity due to the progressive recruitment of neonatal services and changes in prescribing practice over a longer period, such as have already reported others [10].

### Characteristics of the CPOE/CDS system and its reference formulary

Characteristics of the CPOE/CDS system have been previously described [11]. Briefly, this system allows medication prescription according to indication, GA, postnatal age, post-conceptional age, body weight at the day of prescription. The CPOE/CDS system provides a complete prescription made from a reference formulary of 450 Medication INN. The reference formulary is based on French (European) SmPC for licensed medications (http://agence-prd.ansm.sante.fr/php/ecodex). For UOL medications specifications of the Pediatric & Neonatal Dosage Handbook [12] and the medical literature are used as reference. All electronic prescriptions are automatically and totally stored in local computer servers. Monthly they are fully anonymized (de-identification) within each participating hospital before being sent to the same data warehouse for subsequent analyzis. The authorization to do so was given by the National Commission for Data Protection and Privacy (CNIL: DE-2015-099, DE-2017-410) and complies with the most recent French regulation MR-003 which governs research in the health field without obtaining consent [13].

### Recorded data

Data systematically recorded for each prescription have been previously published [11], and are the following:

- Medication INN,
- Date of prescription,
- Indications of medication prescription,
- Dose (international units): unitary dose/kg and inter-dose intervals if discontinuous administration; dose/kg/h or /min if continuous administration; daily dose/kg, loading and maintenance doses when applied (i.e. caffeine, analgesics, antimicrobials),
- Route of administration,
- Preparation modalities with the reconstitution solute, detail of the dilution process when multiple dilutions are required, the volume of rinsing and the volume of perfusion tubing to be added to the total medication volume,
- Report of adverse side effects by prescribers; nine alerts which warn prescribers when an item value is out of the recommended range or when a medication is outside the specifications of the formulary reference or when the body weight has changed by +/- 10%,
- Some clinical information are stored: date of birth, date at admission, gender, gestational age (completed weeks + days), in(out)born status, birth weight and daily bodyweight measures (g), vital status at discharge.

### Non-inclusion criteria

**Fig 1:**
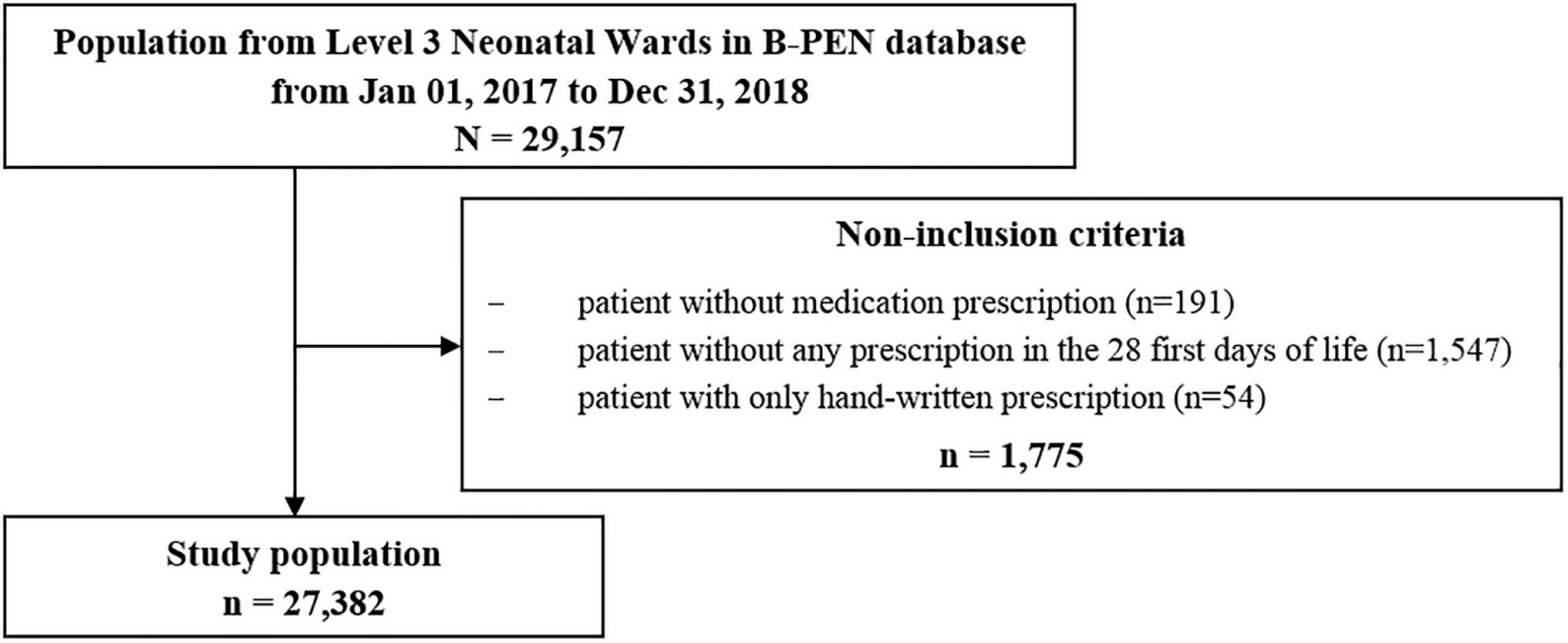
Flow chart diagram from the entire B-PEN program population to the population allowing the study on medication prescriptions in 29 Level 3 Neonatal Wards.

The flow chart (Fig 1) shows how the population of the study was selected. All patients with a first prescription in the 29 L3NW before 28th day of life and at least one electronic medication prescription were eligible to the study.

### Data/Statistical analysis

Pharmaceutical substances were identified by their INN according to WHO general guidance [14] and were categorized according to the Anatomical Therapeutic Chemical (ATC) classification [15]. Three experts (EG, SL, JBG) compared independently each INN to its French SmPC according to the route of administration. The search aimed to identify exposure (INN) and combinations (INN + route) not cited in the first month of life regardless of the indication, dose and frequency of administration.

The national representativeness of the study population was targeted by scrutinizing extremely (GA < 28 weeks of gestation (WG)) and very (28–31 WG) preterm infants since these two categories are constantly cared for in L3NW while this rule does not systematically apply at higher GA. National data about newborns < 32 WG admitted in L3NW were obtained from the French medico-administrative database PMSI (*Programme de Médicalisation des Systèmes d’Information*) which provides information on neonatal care in all public and private French hospitals. The representativeness (% of the total number of national admissions in L3NW) was calculated at each annual quarter of the study since the number of included level 3 hospitals increased gradually from 17 to 29 over the two years of the study.

Results are presented using frequency and proportion for discontinuous variables, and using the mean, standard deviation and extreme values for continuous variables. Khi-square tests and Spearman correlation coefficient are used. Statistical analysis was conducted using SAS^®^ software (Version 9.4, SAS Institute, North Carolina, USA).

## Results

### Level 3 Neonatal Wards

Neonates were included in this retrospective research as soon as their respective L3NW begun to use the CPOE/CDS system of the B-PEN network (Table 1).

**Table 1:** Admissions and number of medication prescriptions in the 29 Level 3 Neonatal Wards of the B-PEN network (2017–2018).

	Level 3 Neonatal Wards		Overall Level 3 Neonatal Wards
	Full period study	Partial period study	
	n = 17	n = 12	n = 29
**Number of year in the study**, mean (SD)	2.0 (0.0)	1.0 (0.5)	1.6 (0.6)
**Number of admissions**, mean (SD)	1220.1 (532.4)	553.3 (434.5)	944.2 (589.7)
[Min–Max]	[618.0–2391.0]	[121.0;1614.0]	[121.0–2391.0]
**Number of monthly admissions**, mean (SD)	50.8 (22.2)	42.1 (16.9)	47.2 (20.3)
[Min–Max]	[25.8–99.6]	[23.2;86.0]	[23.2–99.6]
**Number of different prescribed medication INN**, mean (SD)	113.6 (22.8)	84.5 (21.0)	101.6 (26.1)
[Min–Max]	[76.0–176.0]	[41.0;118.0]	[41.0–176.0]

INN, International non-proprietary name.

**Fig 2:**
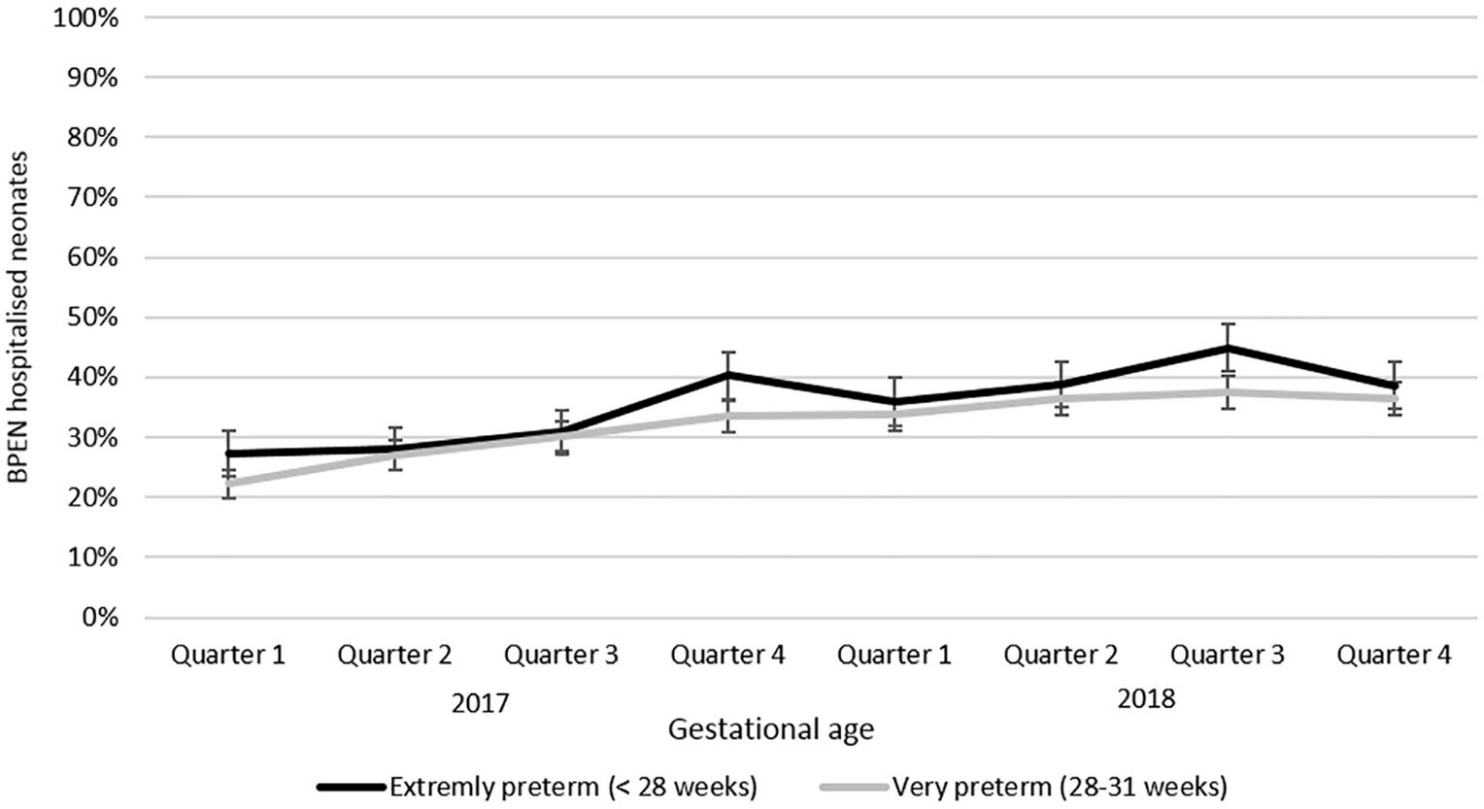
Part of the French extremely and very preterm infants (PMSI data) cared for in the Level 3 Neonatal Wards of the B-PEN network (2017–2018).

The mean contribution of each L3NW to the population of the database was 3.4% (min = 0.4; max = 8.7). Due to the progressive increase in L3NW all along the study period the national representativeness of the study population progressively increased for preterm infants below 32 weeks (Fig 2).

### Population characteristics

Among all hospitalized newborns the main reason of non-inclusion was admission after 28 days of life (5.3% of hospitalized population). Finally, the study population was made up of 27,382 hospitalized neonates with a mean GA of 35.4 (SD = 4.3) weeks and a mean birth weight of 2,458 g (SD = 945). Other demographic characteristics are presented in Table 2.

**Table 2:** Characteristics of the 27,382 neonates cared for in the 29 French Level 3 Neonatal Wards of the B-PEN network (2017–2018).

	Hospitalized neonates n = 27382
**Male**, n (%)	15017 (54.8)
**Multiple pregnancy**, n (%)	2805 (10.2)
**Birth weight (g)**, mean (SD)	2457.8 (944.5)
**Gestational age (weeks)**, mean (SD)	35.4 (4.3)
**Gestational age categories (weeks)**, n (%)	
≤ 27	1740 (6.4)
[28–31]	3293 (12.0)
[32–36]	9350 (34.1)
≥ 37	12999 (47.5)

The mean for estimated length of stay (LOS) was 14.6 days and LOS decreased when GA increased from 47.0 days below 28 WG to 6.4 days above 36 WG (r = -0.55, p < 0.0001) (Table 3). Overall mortality rate was 2.4% and it decreased when GA increased from 17.2% below 28 WG to 1.3% above 36 WG (p < 0.0001) (Table 3).

**Table 3:** Clinical characteristics by gestational age for the 27,382 neonates cared for in the 29 L3NW of the B-PEN program (2017–2018).

	Gestational age (weeks)				Hospitalized neonates
	≤ 27	[28–31]	[32–36]	≥ 37	
	n = 1740	n = 3293	n = 9350	n = 12999	n = 27382
**Length of stay (days)**, mean (SD)	47.0 (38.2)	32.3 (22.7)	13.8 (12.7)	6.4 (8.5)	14.6 (19.5)
**Mortality rate at discharge**, n (%)	299 (17.2)	98 (3.0)	90 (1.0)	174 (1.3)	661 (2.4)
**Postnatal age at first prescription (days)**, mean (SD)	1.7 (5.2)	1.1 (3.9)	1.1 (3.4)	3.3 (6.1)	2.2 (5.1)
**Postnatal age at last prescription (days)**, mean (SD)	47.7 (37.5)	32.4 (22.5)	13.8 (12.9)	8.6 (10.1)	15.8 (19.5)
**Number of prescribed medication INN**, mean (SD)	18.0 (8.2)	11.0 (5.5)	5.4 (3.6)	4.1 (3.5)	6.2 (5.7)
**Neonates with at least one INN drug prescription without any citation in SmPC**, n (%)	1705 (98.0)	3070 (93.2)	6658 (71.2)	5088 (39.1)	16876 (61.6)

INN, International non-proprietary name; SmPC, Summary of product characteristics.

### Characteristics of medication prescription

The mean of medication INN per L3NW was 101.6 (min = 41; max = 176). This number was 113.6 (min = 76; max = 176) for the 17 neonatal wards concerned by the entire study period (Table 1).

Overall the mean number of prescribed medication INN over the hospital stay was 6.2 (SD = 5.7). The mean medication INN number given to neonates in L3NW is significantly increased in preterm infants below 35 WG, the highest exposure being recorded in the extremely preterm neonates (18.0 below 28 WG and 4.1 above 36 WG) (r = -0.54; p < 0.0001).

The mean of postnatal age at first prescription was 2.2 day (SD = 5.1). Overall 78.9% of the first prescription were made in the first two days of life and 88.7% in the first week. The mean of the postnatal age at last prescription was 15.8 (SD = 19.5) days (Table 3).

The leading ATC groups (S1 Table), concerning more than one third of NICU patients, were related to alimentary tract and metabolism (92.6% of neonates), blood and blood forming organs (78.5%), nervous system (56.1%), anti-infective for systemic use (47.6%) and sensory organs (34.4%).

### Exposition to medication INN

Neonatal exposition to medication INN is shown in Tables 4, 5 and 6 and supporting information to these tables in S2, S3 and S4 Tables, respectively.

**Table 4:** Exposed neonates to the 20 medication International Non-proprietary Names most prescribed by gestational age in 29 French Level 3 Neonatal Wards (2017–2018).

	Exposed neonates by gestational age (weeks)				Neonates exposed
	≤ 27	[28–31]	[32–36]	≥ 37	
	n = 1740	n = 3293	n = 9350	n = 12999	n = 27382
**Medication INN prescription**, n (%)					
All Vitamin D	1234 (70.9)	2906 (88.3)	8770 (93.8)	11682 (89.9)	24592 (89.8)
Vitamin D alone	400 (23.0)	955 (29.0)	4627 (49.5)	9096 (70.0)	15078 (55.1)
Vitamin A, D, E, C	1154 (66.3)	2657 (80.7)	5702 (61.0)	3089 (23.8)	12602 (46.0)
Phytomenadione	1673 (96.1)	3170 (96.3)	8217 (87.9)	7795 (60.0)	20855 (76.2)
Paracetamol (acetaminophen)	1141 (65.6)	1566 (47.6)	2669 (28.5)	4733 (36.4)	10109 (36.9)
Gentamicin	1202 (69.1)	1690 (51.3)	2355 (25.2)	3618 (27.8)	8865 (32.4)
Caffeine	1634 (93.9)	3177 (96.5)	2785 (29.8)	233 (1.8)	7829 (28.6)
Cefotaxime	1399 (80.4)	1854 (56.3)	1954 (20.9)	2154 (16.6)	7361 (26.9)
Rifamycin	767 (44.1)	1367 (41.5)	3151 (33.7)	2075 (16.0)	7360 (26.9)
Iron	1053 (60.5)	2323 (70.5)	2825 (30.2)	358 (2.8)	6559 (24.0)
Amoxicillin	537 (30.9)	773 (23.5)	1707 (18.3)	3476 (26.7)	6493 (23.7)
Folic acid	591 (34.0)	1329 (40.4)	1646 (17.6)	276 (2.1)	3842 (14.0)
Amikacin	539 (31.0)	581 (17.6)	700 (7.5)	993 (7.6)	2813 (10.3)
Vancomycin	1069 (61.4)	740 (22.5)	430 (4.6)	513 (3.9)	2752 (10.1)
Sufentanil	753 (43.3)	424 (12.9)	536 (5.7)	913 (7.0)	2626 (9.6)
Midazolam	679 (39.0)	297 (9.0)	460 (4.9)	887 (6.8)	2323 (8.5)
Epoietin alfa	905 (52.0)	1178 (35.8)	133 (1.4)	12 (0.1)	2228 (8.1)
Morphine	667 (38.3)	305 (9.3)	385 (4.1)	835 (6.4)	2192 (8.0)
Atropine	575 (33.0)	511 (15.5)	419 (4.5)	438 (3.4)	1943 (7.1)
Furosemide	769 (44.2)	402 (12.2)	257 (2.7)	470 (3.6)	1898 (6.9)
Tobramycin	294 (16.9)	466 (14.2)	535 (5.7)	320 (2.5)	1615 (5.9)
Ketamine	388 (22.3)	355 (10.8)	326 (3.5)	421 (3.2)	1490 (5.4)

INN, International non-proprietary name

**Table 5:** Exposed neonates to the 20 medication International Non-proprietary Names most prescribed by route of administration in 29 French Level 3 Neonatal Wards (2017–2018).

	Exposed neonates by route of administration n = 27382				
	Injectable	Oral	Respiratory	Ocular / Cutaneous	Others route
**Medication INN prescription**, n (%)					
All Vitamin D	44 (0.2)	24589 (89.8)	-	-	-
Vitamin D alone	44 (0.2)	15045 (54.9)	-	-	-
Vitamin A, D, E, C	-	12602 (46.0)	-	-	-
Phytomenadione	11210 (40.9)	15803 (57.7)	-	-	-
Acetaminophen	6377 (23.3)	6930 (25.3)	-	-	21 (0.1)
Gentamicin	8865 (32.4)	-	-	3 (0.0)	-
Caffeine	7006 (25.6)	5969 (21.8)	-	-	-
Cefotaxime	7361 (26.9)	-	-	-	-
Rifamycin	-	-	-	7360 (26.9)	-
Iron	79 (0.3)	6553 (23.9)	-	-	-
Amoxicillin	6456 (23.6)	159 (0.6)	-	-	-
Folic acid	-	3842 (14.0)	-	-	-
Amikacin	2812 (10.3)	-	-	2 (0.0)	-
Vancomycin	2752 (10.1)	-	-	-	-
Sufentanil	2626 (9.6)	-	-	-	-
Midazolam	2292 (8.4)	17 (0.1)	-	-	62 (0.2)
Epoietin alfa	1089 (4.0)	-	-	-	1708 (6.2)
Morphine	1721 (6.3)	770 (2.8)	-	-	-
Atropine	1938 (7.1)	-	-	10 (0.0)	16 (0.1)
Furosemide	1618 (5.9)	636 (2.3)	-	-	-
Tobramycin	4 (0.0)	-	-	1611 (5.9)	-
Ketamine	1454 (5.3)	-	-	-	54 (0.2)

INN, International non-proprietary name

**Table 6:** The Top 20 medication International Non-proprietary Names without any citation in Summary of Product Characteristics most prescribed by gestational age in 29 French Level 3 Neonatal Wards (2017–2018).

	Exposed neonates to medication INN without any citation in SmPC * by gestational age (weeks)				Neonates exposed medication INN without any citation in SmPC *
	≤ 27	[28–31]	[32–36]	≥ 37	
	n = 1740	n = 3293	n = 9350	n = 12999	n = 27382
**Medication INN prescription**, n (%)					
Rifamycin	767 (44.1)	1367 (41.5)	3151 (33.7)	2075 (16.0)	7360 (26.9)
Acetaminophen	1141 (65.6)	1566 (47.6)	2669 (28.5)	16 (0.1)	5392 (19.7)
Folic acid	591 (34.0)	1329 (40.4)	1646 (17.6)	276 (2.1)	3842 (14.0)
Sufentanil	753 (43.3)	424 (12.9)	536 (5.7)	913 (7.0)	2626 (9.6)
Morphine	667 (38.3)	305 (9.3)	385 (4.1)	835 (6.4)	2192 (8.0)
Atropine	575 (33.0)	511 (15.5)	419 (4.5)	438 (3.4)	1943 (7.1)
Ketamine	388 (22.3)	355 (10.8)	326 (3.5)	421 (3.2)	1490 (5.4)
Lactobacillus	208 (12.0)	461 (14.0)	557 (6.0)	231 (1.8)	1457 (5.3)
Esomeprazole	161 (9.3)	210 (6.4)	338 (3.6)	695 (5.3)	1404 (5.1)
Calcium folinate	194 (11.1)	484 (14.7)	572 (6.1)	54 (0.4)	1304 (4.8)
Glycerin	226 (13.0)	441 (13.4)	401 (4.3)	163 (1.3)	1231 (4.5)
Hydrocortisone	632 (36.3)	177 (5.4)	99 (1.1)	168 (1.3)	1076 (3.9)
Metronidazole	316 (18.2)	264 (8.0)	252 (2.7)	195 (1.5)	1027 (3.8)
Insulin	597 (34.3)	269 (8.2)	69 (0.7)	50 (0.4)	985 (3.6)
Propofol	203 (11.7)	245 (7.4)	224 (2.4)	237 (1.8)	909 (3.3)
Spironolactone	464 (26.7)	300 (9.1)	64 (0.7)	55 (0.4)	883 (3.2)
Sodium alginate and sodium bicarbonate	101 (5.8)	194 (5.9)	229 (2.4)	252 (1.9)	776 (2.8)
Sodium chloride	125 (7.2)	262 (8.0)	220 (2.4)	76 (0.6)	683 (2.5)
Nalbuphine	76 (4.4)	102 (3.1)	165 (1.8)	291 (2.2)	634 (2.3)
Budesonide	335 (19.3)	147 (4.5)	41 (0.4)	109 (0.8)	632 (2.3)

INN, International non-proprietary name; SmPC, Summary of product characteristics.

* without any citation for the combination of the medication INN and its route of administration

Overall 261 different medication INN were prescribed (Tables 3 and 4 and S2 Table):

- 12 medication INN to at least 10% of the patients: vitamin D (89.8%) given alone (55.1%) or in association with vitamins A, E, C (46.0%), phytomenadione (76.2%), paracetamol (i.e. acetaminophen) (36.9%), gentamicin (32.4%), caffeine (28.6%), cefotaxime (26.9%), rifamycin by ocular route (26.9%), iron (24.0%), amoxicillin (23.7%), folic acid (14.0%), amikacin (10.3%) and vancomycin (10.1%);
- 55 medication INN were administrated to less than 10% of patients but at least 1%;
- 194 medication INN (74% of medications) were administrated to less than 1% of patients each.

Table 4 shows the exposure to each of the 20 most prescribed medication INN at each GA group. Among these 20 leading medications 7 are antibiotics, 5 are analgesic or sedative medications and 5 are in the field of nutrition-metabolism.

Examining the 67 most prescribed medications (S2 Table) the group with GA below 28 WG presented the highest exposure rate for 55 INN (82.1%). The 28–31 weeks group presented the highest exposure rate for 9 INN (11.9%) and the second highest exposure rate for 51 INN (71.6%).

Patients were prescribed 351 different combinations of medication INN and routes of administration of which 149 were injectable and 126 were oral (Table 5 and S3 Table). Other less frequent routes were respiratory (n = 9), cutaneous (n = 20), ocular (n = 22) and various (n = 25). Among the 351 combinations (INN by route of administration), 243 (69.2%) were not cited for use in the first month of life according to the French SmPC.

Overall 61.6% of neonates were prescribed at least one medication INN without any citation in SmPC for prescription in the first month of life. The highest rate was recorded in the extremely preterm neonates (98.0% below 28 WG to 39.1% above 37 WG) (p < 0.0001) (Table 3). Among INN prescribed without any SmPC (Table 6 and S4 Table), the 3 most prescribed were rifamycin (ocular) for 26.9% of treated neonates, paracetamol (i.e. acetaminophen) for 19.7% and folic acid for 14.0% (Table 6).

## Discussion

This retrospective observational study seeks to provide better knowledge of the medications prescribed in L3NW. It is based on recent data collected over a 2-year period from 27,382 neonates hospitalized in 29 of the 68 French L3NW (42.6%). Overall a limited number of medication INN were prescribed with an unequal distribution among neonates since only twelve of the 261 (4.60%) medication INN were given to more than 10% of the population and 194 (74.3%) to less than 1%. Medication INN not cited in SmPC represented 69.2% of medication prescriptions. The most immature infants (GA < 32 WG) were exposed to the higher rates for both total and not cited medication INN.

Comparison to other studies on medications use in NICU is done through both recent extensive systematic reviews of these studies [2–4] and some large epidemiological studies [10].

Compared to other studies, this cohort is characterized by the fact that all L3NW participate in the same program of comparative analysis of their prescriptions (B-PEN program) and the study describes the initial situation before any structured action to modify prescribing practices.

The cornerstone of the B-PEN program is a CPOE / CDS system implemented in all L3NW involved in the program. It is powered by a reference therapeutic formulary. This formulary is not totally mandatory for the teams as it allows some modifications in a structured and controlled manner to fit at best with local therapeutic habits. Each month, a complete anonymization (deidentification) of electronic prescription files is performed locally in each participating hospital before sending the prescriptions to a common data warehouse [11].

The 261 prescribed medication INN are fewer as compared to the 409 medications recorded in the administrative data base of the Pediatrix Medical Group in 2006 [16] but this number is higher than the 229 medications reported in the same hospital network in 2014 [10]. This number is also well below the 450 medication INN of the general reference formulary initially proposed to each center and the 1008 generic medications recently identified in 42 US pediatric intensive care units [17].

This French cohort was similar to the Pediatrix one [10] for the mean number of medications given to the overall population (n = 4) and the huge mean number recorded in both US ELBW neonates (mean = 17) and in French extremely preterm neonates (mean = 18). Overall extremely preterm infants are exposed to six times more medication INN than at term newborns cared for in L3NW.

This trend is probably universal since it was also recently observed in Italy, Brazil and Germany [18–20] and this study adds precision showing that almost all medications are concerned by a preeminent use in the preterm neonates born before 32 WG as they have the highest exposure rate for 64 of the 67 most prescribed medications in L3NW.

This study also shows large variations in medication exposure rate (from 76 to 176) between the 17 L3NW which were included at the onset of this study and were fully and similarly observed over a 2 year-period. A similar variability was recently highlighted between four Dutch NICU for a total of 181 different medication INN [21]. A large part of studies agree on the variability in the medication prescription between centers [2–4] but also between countries [22]. Explaining the variability between L3NW is out of the scope of this study but it is worth noting that this variability is observed while the same CPOE system was used everywhere. Thus the variability may rely on differences between neonatal populations in L3NW and/or a lack of guidelines thus giving a large place to local experience and local therapeutic habits.

Even if a high exposure rate (above 10% of patients) is limited to 12 medication INN, six of them are antibiotics either systemic (amoxicilline, cefotaxime, gentamicin, amikacine, vancomycin) or ocular (rifamycin). The most commonly used medications in this French serie are close to findings in 19 other studies performed over 12 countries [1–4]. For instance the “Top Ten” of the most prescribed medication INN is partially shared with the Netherlands [21], i.e.: phytomenadione (vitamin K), cholecalciferol (vitamin D), caffeine, amoxicillin, gentamicin, paracetamol (i.e. acetaminophen). However the last large US study only found ampicillin, gentamicin and vancomycin in the group of the most prescribed medications [10].

Given the universally unfavorable evolution of antibiotic resistance and the harmful consequences of antibiotic pressure on the spectrum of neonatal diseases [23], the monitoring of antibiotic therapy in all L3NW is an effort that have proved ultimately productive by the effectiveness of the resulting preventive actions [24].

Practically this study points out a high prescription rate of cefotaxime in very preterm infants (80.4% below 28 WG, 56.3% at 28–31 weeks; 20.9% at 32–36 weeks and 16.6% above 36 weeks) thus suggesting a high level of bacteriologically Gram negative sepsis. However Puopolo et al. [25] recently showed that the group of US ELBW infants (22–28 WG) at high risk of early onset sepsis (EOS) had a bacteriologically proven infection rate as low as 2.5%. If the trend in Gram negative EOS is similar in France a limitation of cefotaxime consumption in the French L3NW will be a challenge. Seeing that the B-PEN network represent 42.6% of French NICU in 2018 as well as 40% of hospital admissions of preterm infants below 32 WG, a continuous monitoring of antibiotics will have to be enlarged both quantitatively and qualitatively (more clinical and bacteriological data) for an optimized guidance of antibiotics strategy in neonatal wards.

The high prevalence of paracetamol prescription in this study exemplifies an unexpected new trend as it ranked 3rd and was prescribed to 36.9% of patients. This trend was lacking in the recent Krzyzaniak’s literature reviews [1–4] as paracetamol was not among the 20 most commonly medications prescribed in NICU. A specific study on paracetamol in French L3NW is all the more necessary as the highest prescription rate is observed in preterm infants particularly below 32 WG whereas the French SmPC for paracetamol limits its administration to term babies. It is worth noting that the rate of paracetamol prescription is not equivalent to its rate of administration which can depends on the results of pain scores assessment.

It is recognized that NICU patients mainly receive UOL medications, the highest incidence being in the most immature infants. Whatever the country the risk of exposure to an unlicensed medication ranged from 11% to 69% and from 33% to 96% for off-label medications [18–20, 26–32].

This study shows that even ignoring the medication’s indication, dose and frequency of administration, but only considering the route of administration for each medication INN, 69.2% of prescribed combinations are not cited in the SmPC for use in the first month of life thus making them automatically UOL. In the years 2010 a similar result (62%) has been found in Germany [20]. This study also shows that the risk of being prescribed a not-cited medication exceeds 95% in premature babies under 32 WG.

This research method aims promotion of neonatal pharmacoepidemiology with some strengths and weaknesses. The database fits well with the setting up of a neonatal prescriptions registry as a witness of evidence in the real world practice. Moreover the constant recording of prescriptions is the prerequisite of a long term benchmarking of neonatal wards. Large representation of the population also offers possibilities for public health surveys.

The question of representativeness is important but will need further specific approaches. Indeed it can be suggested that hospitalized patients in the 68 French L3NW are effectively mirrored by the 29 L3NW (42.6%) of the B-PEN program that includes about 40% of the French newborns under 32 WG. The overall French population was identified via a national medico-administrative database (*Programme de Médicalisation des Systèmes d’Information*, PMSI) whose validity for GA has been previously shown satisfactory [33].

The main limit relies on the lack of therapeutic information after the neonatal hospitalization but also before admission in L3NW (including antenatal life and care in the delivery room). This point is well exemplified by surfactant treatment which is given to 96.3% at 22–26 WG and 58.7% at 27–31 WG in the French national epidemiological study EPIPAGE 2 [34]. However this study indicates lower exposure rates to surfactant in the L3NW (31.6% at 22–27 WG and 17.9% at 28–31 WG). Since most of the first dose of surfactant is given in the delivery room in France [34], this good clinical practice [35] explains the low incidence of surfactant administration in L3NW.

Finally, some prescriptions were handwritten (typed without any help of the CPOE) and their dosing and administration route have been electronically stored and not included in this study. They have been evaluated at 3% of prescriptions in the B-PEN data base.

## Conclusion

This study shows the feasibility of a continuous record of all medications prescribed in L3NW. It arises the question of polypharmacy and potential increased medication-medication interactions in NICU, particularly in the most immature neonates [17]. The intense exposition of the preterm infants to pharmacological agents will require sufficient consideration in future studies on their short- and long-term consequences. Since neonates remain therapeutic orphans, in depth therapeutic database represents a major challenge for the health of the most immature neonates.

## Supporting information

S1 Table The Anatomical Therapeutic Chemical classification system for medications prescribed to 27,382 neonates cared for in 29 French Level 3 Neonatal Wards (2017–2018). (DOCX)

S2 Table Exposed neonates to the less prescribed medication International Non-Proprietary Names by gestational age in 29 French Level 3 Neonatal Wards (2017–2018). (DOCX)

S3 Table Exposed neonates to the less prescribed medication International Non-Proprietary Names by route of administration in 29 French Level 3 Neonatal Wards (2017–2018). (DOCX)

S4 Table Exposed neonates to the less prescribed medication International Non-Proprietary Names without any citation in Summary of Product Characteristics by gestational age in 29 French Level 3 Neonatal Wards (2017–2018). (DOCX)

S1 Data Admission and medication INN prescription by hospital. (XLSX)

S2 Data Demographical and clinical patient characteristics. (XLSX)

S3 Data Exposed neonates to medication INN. (XLSX)

S4 Data Exposed neonates to injectable medication INN. (XLSX)

S5 Data Exposed neonates to oral medication INN. (XLSX)

S6 Data Exposed neonates to respiratory medication INN. (XLSX)

S7 Data Exposed neonates to ocular or cutaneous medication INN. (XLSX)

S8 Data Exposed neonates to other administration route medication INN. (XLSX)

S9 Data Exposed neonates to medication INN without any citation in SmPC. (XLSX)
