# Supplementary material for: Characteristics of prescription in 29 Level 3 Neonatal Wards over a 2-year period (2017-2018). An inventory for future research
Source: PLoS One. 2019 Sep 19;14(9):e0222667. doi: 10.1371/journal.pone.0222667 (PMC6752821; doi:10.1371/journal.pone.0222667)
Supplement: S1 Table — (DOCX) [file pone.0222667.s001.docx]

**S1 Table**. The Anatomical Therapeutic Chemical classification system for medications prescribed to 27,382 neonates cared for in 29 French Level 3 Neonatal Wards (2017-2018)

|  | **Neonates exposed** |
| --- | --- |
|  | **n=27382** |
| **ATC classification of medication prescription**, n (%) |  |
| A - Alimentary tract and metabolism | 25362    (92.6) |
| B - Blood and blood forming organs | 21499    (78.5) |
| N - Nervous system | 15352    (56.1) |
| J - General antiinfectives for systemic use | 13022    (47.6) |
| S - Sensory organs | 9406    (34.4) |
| C - Cardiovascular system | 3708    (13.5) |
| R - Respiratory system | 2388      (8.7) |
| V - Various | 1613      (5.9) |
| H - Systemic hormonal prep, excluding sex hormones | 1540      (5.6) |
| D - Dermatologicals | 1341      (4.9) |
| M - Musculo-skeletal system | 322      (1.2) |
| L - Antineoplastic and immunomodulating agents | 108      (0.4) |
| G - Genito urinary system and sex hormones | 92      (0.3) |
| P - Antiparasitic products | 12      (0.0) |

ATC, anatomical therapeutic chemical
