# Supplementary material for: Characteristics of prescription in 29 Level 3 Neonatal Wards over a 2-year period (2017-2018). An inventory for future research
Source: PLoS One. 2019 Sep 19;14(9):e0222667. doi: 10.1371/journal.pone.0222667 (PMC6752821; doi:10.1371/journal.pone.0222667)
Supplement: S2 Table — (DOCX) [file pone.0222667.s002.docx]

**S2 Table**. Exposed neonates to the less prescribed medication International Non-Proprietary Names by gestational age in 29 French Level 3 Neonatal Wards (2017-2018)

|  | **Exposed neonates by gestational age (weeks)** | | | | **Neonates** |
| --- | --- | --- | --- | --- | --- |
|  | **≤ 27** | **[28 - 31]** | **[32 - 36]** | **≥ 37** | **exposed** |
|  | **n = 1740** | **n = 3293** | **n = 9350** | **n = 12999** | **n=27382** |
| **Medication INN prescription**, n (%) |  |  |  |  |  |
| Lactobacillus | 208 (12.0) | 461 (14.0) | 557 (6.0) | 231 (1.8) | 1457 (5.3) |
| Poractant alfa | 523 (30.1) | 536 (16.3) | 250 (2.7) | 108 (0.8) | 1417 (5.2) |
| Esomeprazole | 161 (9.3) | 210 (6.4) | 338 (3.6) | 695 (5.3) | 1404 (5.1) |
| Sodium chloride | 310 (17.8) | 386 (11.7) | 357 (3.8) | 316 (2.4) | 1369 (5.0) |
| Calcium folinate | 194 (11.1) | 484 (14.7) | 572 (6.1) | 54 (0.4) | 1304 (4.8) |
| Glycerin | 226 (13.0) | 441 (13.4) | 401 (4.3) | 163 (1.3) | 1231 (4.5) |
| Fluconazole | 816 (46.9) | 313 (9.5) | 47 (0.5) | 53 (0.4) | 1229 (4.5) |
| Hydrocortisone | 632 (36.3) | 177 (5.4) | 99 (1.1) | 168 (1.3) | 1076 (3.9) |
| Vaccine pneumococcal polysaccharide conjugates 13-valent absorbed | 443 (25.5) | 486 (14.8) | 87 (0.9) | 24 (0.2) | 1040 (3.8) |
| Metronidazole | 316 (18.2) | 264 (8.0) | 252 (2.7) | 195 (1.5) | 1027 (3.8) |
| Vaccine diphtheria, tetanus, acellular pertussis, hepatitis B, inactive poliomyelitis and haemophilus influenzae type B conjugate | 421 (24.2) | 463 (14.1) | 79 (0.8) | 26 (0.2) | 989 (3.6) |
| Insulin | 597 (34.3) | 269 (8.2) | 69 (0.7) | 50 (0.4) | 985 (3.6) |
| Dopamine | 550 (31.6) | 166 (5.0) | 102 (1.1) | 116 (0.9) | 934 (3.4) |
| Nystatin | 141 (8.1) | 200 (6.1) | 238 (2.5) | 336 (2.6) | 915 (3.3) |
| Norepinephrine tartrate | 328 (18.9) | 140 (4.3) | 132 (1.4) | 312 (2.4) | 912 (3.3) |
| Propofol | 203 (11.7) | 245 (7.4) | 224 (2.4) | 237 (1.8) | 909 (3.3) |
| Spironolactone | 464 (26.7) | 300 (9.1) | 64 (0.7) | 55 (0.4) | 883 (3.2) |
| Econazole | 230 (13.2) | 236 (7.2) | 247 (2.6) | 144 (1.1) | 857 (3.1) |
| Ibuprofen | 574 (33.0) | 216 (6.6) | 11 (0.1) | 1 (0.0) | 802 (2.9) |
| Sodium alginate and sodium bicarbonate | 101 (5.8) | 194 (5.9) | 229 (2.4) | 252 (1.9) | 776 (2.8) |
| Palivizumab | 276 (15.9) | 359 (10.9) | 79 (0.8) | 28 (0.2) | 742 (2.7) |
| Albumin | 214 (12.3) | 171 (5.2) | 164 (1.8) | 191 (1.5) | 740 (2.7) |
| Nalbuphine | 76 (4.4) | 102 (3.1) | 165 (1.8) | 291 (2.2) | 634 (2.3) |
| Budesonide | 335 (19.3) | 147 (4.5) | 41 (0.4) | 109 (0.8) | 632 (2.3) |
| Lidocaine and prilocaine | 173 (9.9) | 247 (7.5) | 79 (0.8) | 58 (0.4) | 557 (2.0) |
| Picloxydine | 54 (3.1) | 118 (3.6) | 180 (1.9) | 166 (1.3) | 518 (1.9) |
| Phenobarbital | 48 (2.8) | 26 (0.8) | 76 (0.8) | 362 (2.8) | 512 (1.9) |
| Tropicamide | 188 (10.8) | 215 (6.5) | 59 (0.6) | 44 (0.3) | 506 (1.8) |
| Fentanyl | 150 (8.6) | 80 (2.4) | 95 (1.0) | 157 (1.2) | 482 (1.8) |
| Alfacalcidol | 20 (1.1) | 40 (1.2) | 281 (3.0) | 107 (0.8) | 448 (1.6) |
| Heparin | 85 (4.9) | 34 (1.0) | 69 (0.7) | 225 (1.7) | 413 (1.5) |
| Calcium gluconate | 50 (2.9) | 54 (1.6) | 209 (2.2) | 98 (0.8) | 411 (1.5) |
| Recombinant hepatitis B vaccine adsorbed | 17 (1.0) | 39 (1.2) | 198 (2.1) | 153 (1.2) | 407 (1.5) |
| Betamethasone | 266 (15.3) | 42 (1.3) | 25 (0.3) | 70 (0.5) | 403 (1.5) |
| Phenylephrine | 163 (9.4) | 169 (5.1) | 40 (0.4) | 30 (0.2) | 402 (1.5) |
| Dobutamine | 110 (6.3) | 60 (1.8) | 61 (0.7) | 163 (1.3) | 394 (1.4) |
| Sodium bicarbonate | 235 (13.5) | 55 (1.7) | 39 (0.4) | 65 (0.5) | 394 (1.4) |
| Epinephrine | 119 (6.8) | 49 (1.5) | 56 (0.6) | 166 (1.3) | 390 (1.4) |
| Pantoprazole | 52 (3.0) | 38 (1.2) | 96 (1.0) | 199 (1.5) | 385 (1.4) |
| Amphotericin B | 85 (4.9) | 105 (3.2) | 91 (1.0) | 71 (0.5) | 352 (1.3) |
| Alprostadil | 5 (0.3) | 4 (0.1) | 50 (0.5) | 246 (1.9) | 305 (1.1) |
| Atracurium besilate | 113 (6.5) | 30 (0.9) | 35 (0.4) | 114 (0.9) | 292 (1.1) |
| Piperacillin and tazobactam | 106 (6.1) | 45 (1.4) | 66 (0.7) | 72 (0.6) | 289 (1.1) |
| Azithromycin | 53 (3.0) | 41 (1.2) | 88 (0.9) | 96 (0.7) | 278 (1.0) |
| Mupirocin | 136 (7.8) | 86 (2.6) | 25 (0.3) | 21 (0.2) | 268 (1.0) |
| Meropenem | 139 (8.0) | 68 (2.1) | 35 (0.4) | 25 (0.2) | 267 (1.0) |
| Cefepime | 117 (6.7) | 60 (1.8) | 35 (0.4) | 50 (0.4) | 262 (1.0) |
| Salbutamol | 144 (8.3) | 30 (0.9) | 26 (0.3) | 50 (0.4) | 250 (0.9) |
| Ceftazidime | 145 (8.3) | 53 (1.6) | 21 (0.2) | 23 (0.2) | 242 (0.9) |
| Dimeticone | 10 (0.6) | 5 (0.2) | 98 (1.0) | 127 (1.0) | 240 (0.9) |
| Acyclovir | 5 (0.3) | 11 (0.3) | 27 (0.3) | 196 (1.5) | 239 (0.9) |
| Ranitidine | 73 (4.2) | 63 (1.9) | 35 (0.4) | 56 (0.4) | 227 (0.8) |
| nitric oxide | 74 (4.3) | 28 (0.9) | 31 (0.3) | 72 (0.6) | 205 (0.7) |
| Phosphore element | 72 (4.1) | 78 (2.4) | 37 (0.4) | 15 (0.1) | 202 (0.7) |
| Amoxicillin and clavulanate | 9 (0.5) | 14 (0.4) | 37 (0.4) | 132 (1.0) | 192 (0.7) |
| Phenytoin | 9 (0.5) | 5 (0.2) | 29 (0.3) | 145 (1.1) | 188 (0.7) |
| Levocarnitine | 37 (2.1) | 74 (2.2) | 42 (0.4) | 32 (0.2) | 185 (0.7) |
| Cefazolin | 36 (2.1) | 9 (0.3) | 48 (0.5) | 90 (0.7) | 183 (0.7) |
| Ursodesoxycholic acid | 66 (3.8) | 51 (1.5) | 30 (0.3) | 26 (0.2) | 173 (0.6) |
| Zinc oxide | 20 (1.1) | 35 (1.1) | 84 (0.9) | 32 (0.2) | 171 (0.6) |
| Fluticasone | 111 (6.4) | 37 (1.1) | 8 (0.1) | 13 (0.1) | 169 (0.6) |
| Dexpanthenol | 33 (1.9) | 46 (1.4) | 67 (0.7) | 16 (0.1) | 162 (0.6) |
| Naloxone | 74 (4.3) | 17 (0.5) | 29 (0.3) | 41 (0.3) | 161 (0.6) |
| Oxacillin | 75 (4.3) | 31 (0.9) | 17 (0.2) | 38 (0.3) | 161 (0.6) |
| Tuberculosis vaccine | 5 (0.3) | 13 (0.4) | 87 (0.9) | 55 (0.4) | 160 (0.6) |
| Doxapram | 113 (6.5) | 36 (1.1) | 1 (0.0) | 0 (0.0) | 150 (0.5) |
| Josamycin | 91 (5.2) | 33 (1.0) | 5 (0.1) | 17 (0.1) | 146 (0.5) |
| Zidovudine | 9 (0.5) | 11 (0.3) | 36 (0.4) | 86 (0.7) | 142 (0.5) |
| Piperacillin | 69 (4.0) | 40 (1.2) | 15 (0.2) | 15 (0.1) | 139 (0.5) |
| Retinol | 58 (3.3) | 29 (0.9) | 15 (0.2) | 35 (0.3) | 137 (0.5) |
| Glucagon | 2 (0.1) | 7 (0.2) | 56 (0.6) | 63 (0.5) | 128 (0.5) |
| Ornidazole | 32 (1.8) | 15 (0.5) | 33 (0.4) | 43 (0.3) | 123 (0.4) |
| Sorbitol | 27 (1.6) | 50 (1.5) | 33 (0.4) | 13 (0.1) | 123 (0.4) |
| Diazepam | 8 (0.5) | 6 (0.2) | 23 (0.2) | 85 (0.7) | 122 (0.4) |
| Ciclopirox | 13 (0.7) | 26 (0.8) | 43 (0.5) | 38 (0.3) | 120 (0.4) |
| Levetiracetam | 6 (0.3) | 7 (0.2) | 11 (0.1) | 96 (0.7) | 120 (0.4) |
| Imipenem and cilastatin | 66 (3.8) | 23 (0.7) | 9 (0.1) | 21 (0.2) | 119 (0.4) |
| Ciprofloxacin | 33 (1.9) | 27 (0.8) | 16 (0.2) | 39 (0.3) | 115 (0.4) |
| Anti-hepatitis B immune globulin | 14 (0.8) | 21 (0.6) | 45 (0.5) | 30 (0.2) | 110 (0.4) |
| Filgrastim | 50 (2.9) | 48 (1.5) | 6 (0.1) | 4 (0.0) | 108 (0.4) |
| Hydroxyzine | 19 (1.1) | 8 (0.2) | 11 (0.1) | 59 (0.5) | 97 (0.4) |
| Ceftriaxone | 3 (0.2) | 6 (0.2) | 14 (0.1) | 73 (0.6) | 96 (0.4) |
| Clonazepam | 7 (0.4) | 6 (0.2) | 12 (0.1) | 71 (0.5) | 96 (0.4) |
| Folinic acid | 10 (0.6) | 32 (1.0) | 49 (0.5) | 2 (0.0) | 93 (0.3) |
| Micafungin | 73 (4.2) | 15 (0.5) | 2 (0.0) | 2 (0.0) | 92 (0.3) |
| Sildenafil | 18 (1.0) | 9 (0.3) | 11 (0.1) | 54 (0.4) | 92 (0.3) |
| Immune glogulin | 2 (0.1) | 7 (0.2) | 20 (0.2) | 62 (0.5) | 91 (0.3) |
| Erythromycin | 31 (1.8) | 45 (1.4) | 10 (0.1) | 4 (0.0) | 90 (0.3) |
| Levothyroxine sodium | 15 (0.9) | 7 (0.2) | 30 (0.3) | 38 (0.3) | 90 (0.3) |
| Cefaclor | 1 (0.1) | 3 (0.1) | 17 (0.2) | 68 (0.5) | 89 (0.3) |
| Milrinone | 17 (1.0) | 7 (0.2) | 14 (0.1) | 51 (0.4) | 89 (0.3) |
| Linezolid | 55 (3.2) | 13 (0.4) | 7 (0.1) | 11 (0.1) | 86 (0.3) |
| Vaccine diphtheria, tetanus, acellular pertussis, inactive poliomyelitis and haemophilus influenzae type B conjugate | 52 (3.0) | 26 (0.8) | 5 (0.1) | 0 (0.0) | 83 (0.3) |
| Omeprazole | 9 (0.5) | 5 (0.2) | 20 (0.2) | 47 (0.4) | 81 (0.3) |
| Amiodarone | 2 (0.1) | 4 (0.1) | 22 (0.2) | 39 (0.3) | 67 (0.2) |
| Glucose | 18 (1.0) | 10 (0.3) | 23 (0.2) | 14 (0.1) | 65 (0.2) |
| Carbomer | 9 (0.5) | 3 (0.1) | 11 (0.1) | 39 (0.3) | 62 (0.2) |
| Bumetanide | 23 (1.3) | 10 (0.3) | 7 (0.1) | 13 (0.1) | 53 (0.2) |
| Benzylpenicillin | 1 (0.1) | 3 (0.1) | 7 (0.1) | 41 (0.3) | 52 (0.2) |
| Amphotericin B liposomal | 41 (2.4) | 8 (0.2) | 1 (0.0) | 1 (0.0) | 51 (0.2) |
| Clonidine | 25 (1.4) | 7 (0.2) | 7 (0.1) | 12 (0.1) | 51 (0.2) |
| Tocofersolan | 22 (1.3) | 13 (0.4) | 8 (0.1) | 8 (0.1) | 51 (0.2) |
| Adenosine phosphate | 1 (0.1) | 4 (0.1) | 18 (0.2) | 27 (0.2) | 50 (0.2) |
| Biotin | 1 (0.1) | 2 (0.1) | 10 (0.1) | 36 (0.3) | 49 (0.2) |
| Methylprednisolone | 7 (0.4) | 4 (0.1) | 9 (0.1) | 29 (0.2) | 49 (0.2) |
| Lamivudine | 2 (0.1) | 3 (0.1) | 7 (0.1) | 36 (0.3) | 48 (0.2) |
| Pyridoxine | 1 (0.1) | 2 (0.1) | 4 (0.0) | 41 (0.3) | 48 (0.2) |
| Rifampicin | 15 (0.9) | 13 (0.4) | 5 (0.1) | 15 (0.1) | 48 (0.2) |
| Hydrochlorothiazide | 32 (1.8) | 13 (0.4) | 1 (0.0) | 0 (0.0) | 46 (0.2) |
| Propranolol | 2 (0.1) | 4 (0.1) | 15 (0.2) | 25 (0.2) | 46 (0.2) |
| Fusidic acid | 13 (0.7) | 9 (0.3) | 9 (0.1) | 13 (0.1) | 44 (0.2) |
| Epoprostenol | 6 (0.3) | 7 (0.2) | 5 (0.1) | 24 (0.2) | 42 (0.2) |
| Nicardipine | 8 (0.5) | 16 (0.5) | 5 (0.1) | 11 (0.1) | 40 (0.1) |
| Aluminium phosphate | 1 (0.1) | 4 (0.1) | 21 (0.2) | 13 (0.1) | 39 (0.1) |
| Nevirapine | 1 (0.1) | 2 (0.1) | 6 (0.1) | 30 (0.2) | 39 (0.1) |
| Bifonazole | 5 (0.3) | 4 (0.1) | 17 (0.2) | 11 (0.1) | 37 (0.1) |
| Sodium polystyrene sulfonate | 5 (0.3) | 4 (0.1) | 10 (0.1) | 18 (0.1) | 37 (0.1) |
| Suxamethonium | 11 (0.6) | 4 (0.1) | 9 (0.1) | 12 (0.1) | 36 (0.1) |
| valproic acid | 2 (0.1) | 1 (0.0) | 3 (0.0) | 29 (0.2) | 35 (0.1) |
| Diosmectite | 10 (0.6) | 5 (0.2) | 12 (0.1) | 7 (0.1) | 34 (0.1) |
| Thiamine | 0 (0.0) | 3 (0.1) | 7 (0.1) | 24 (0.2) | 34 (0.1) |
| Domperidone | 9 (0.5) | 10 (0.3) | 8 (0.1) | 5 (0.0) | 32 (0.1) |
| Teicoplanin | 14 (0.8) | 9 (0.3) | 2 (0.0) | 7 (0.1) | 32 (0.1) |
| Sulfamethoxazole and trimethoprim | 6 (0.3) | 3 (0.1) | 6 (0.1) | 16 (0.1) | 31 (0.1) |
| Oxiconazole | 3 (0.2) | 10 (0.3) | 9 (0.1) | 8 (0.1) | 30 (0.1) |
| Trimebutine | 5 (0.3) | 6 (0.2) | 6 (0.1) | 13 (0.1) | 30 (0.1) |
| Adenosine | 1 (0.1) | 3 (0.1) | 5 (0.1) | 19 (0.1) | 28 (0.1) |
| Lidocaine | 2 (0.1) | 3 (0.1) | 4 (0.0) | 19 (0.1) | 28 (0.1) |
| Clindamycin | 14 (0.8) | 5 (0.2) | 3 (0.0) | 4 (0.0) | 26 (0.1) |
| Digoxin | 0 (0.0) | 0 (0.0) | 8 (0.1) | 18 (0.1) | 26 (0.1) |
| Sodium benzoate | 1 (0.1) | 1 (0.0) | 3 (0.0) | 21 (0.2) | 26 (0.1) |
| Cefixime | 4 (0.2) | 1 (0.0) | 4 (0.0) | 16 (0.1) | 25 (0.1) |
| Acebutolol | 2 (0.1) | 0 (0.0) | 10 (0.1) | 12 (0.1) | 24 (0.1) |
| Thiopental sodium | 1 (0.1) | 3 (0.1) | 6 (0.1) | 13 (0.1) | 23 (0.1) |
| Captopril | 1 (0.1) | 2 (0.1) | 8 (0.1) | 11 (0.1) | 22 (0.1) |
| Diazoxide | 0 (0.0) | 2 (0.1) | 4 (0.0) | 16 (0.1) | 22 (0.1) |
| Riboflavin | 0 (0.0) | 3 (0.1) | 6 (0.1) | 13 (0.1) | 22 (0.1) |
| Ganciclovir | 8 (0.5) | 3 (0.1) | 4 (0.0) | 6 (0.0) | 21 (0.1) |
| Oseltamivir | 2 (0.1) | 5 (0.2) | 4 (0.0) | 10 (0.1) | 21 (0.1) |
| Zinc | 10 (0.6) | 6 (0.2) | 4 (0.0) | 1 (0.0) | 21 (0.1) |
| Hyaluronic acid | 8 (0.5) | 6 (0.2) | 4 (0.0) | 1 (0.0) | 19 (0.1) |
| Silver nitrate | 2 (0.1) | 7 (0.2) | 4 (0.0) | 5 (0.0) | 18 (0.1) |
| Terbutaline | 6 (0.3) | 1 (0.0) | 3 (0.0) | 8 (0.1) | 18 (0.1) |
| Acetylsalicylic acid | 0 (0.0) | 1 (0.0) | 3 (0.0) | 13 (0.1) | 17 (0.1) |
| Carmellose | 2 (0.1) | 0 (0.0) | 1 (0.0) | 14 (0.1) | 17 (0.1) |
| Prednisolone | 2 (0.1) | 1 (0.0) | 4 (0.0) | 10 (0.1) | 17 (0.1) |
| Valganciclovir | 5 (0.3) | 3 (0.1) | 2 (0.0) | 7 (0.1) | 17 (0.1) |
| Cefamandole | 5 (0.3) | 1 (0.0) | 6 (0.1) | 4 (0.0) | 16 (0.1) |
| Alimemazine | 5 (0.3) | 1 (0.0) | 1 (0.0) | 8 (0.1) | 15 (0.1) |
| Desomedine | 1 (0.1) | 3 (0.1) | 9 (0.1) | 2 (0.0) | 15 (0.1) |
| Sulfadiazine | 3 (0.2) | 0 (0.0) | 2 (0.0) | 10 (0.1) | 15 (0.1) |
| Theophylline | 7 (0.4) | 1 (0.0) | 2 (0.0) | 3 (0.0) | 13 (0.0) |
| Arginine | 0 (0.0) | 1 (0.0) | 1 (0.0) | 10 (0.1) | 12 (0.0) |
| Flumazenil | 8 (0.5) | 0 (0.0) | 0 (0.0) | 4 (0.0) | 12 (0.0) |
| Miconazole | 0 (0.0) | 1 (0.0) | 3 (0.0) | 8 (0.1) | 12 (0.0) |
| Pyrimethamine | 0 (0.0) | 0 (0.0) | 1 (0.0) | 11 (0.1) | 12 (0.0) |
| Cethexonium bromide | 1 (0.1) | 2 (0.1) | 5 (0.1) | 3 (0.0) | 11 (0.0) |
| Norfloxacin | 4 (0.2) | 2 (0.1) | 2 (0.0) | 3 (0.0) | 11 (0.0) |
| Antithrombin III | 0 (0.0) | 1 (0.0) | 2 (0.0) | 7 (0.1) | 10 (0.0) |
| Fosfomycin | 4 (0.2) | 4 (0.1) | 0 (0.0) | 2 (0.0) | 10 (0.0) |
| Amitriptyline | 0 (0.0) | 0 (0.0) | 1 (0.0) | 8 (0.1) | 9 (0.0) |
| Fludrocortisone | 1 (0.1) | 2 (0.1) | 2 (0.0) | 4 (0.0) | 9 (0.0) |
| Ofloxacin | 0 (0.0) | 4 (0.1) | 3 (0.0) | 2 (0.0) | 9 (0.0) |
| Phenoxymethyl penicillin | 0 (0.0) | 0 (0.0) | 3 (0.0) | 6 (0.0) | 9 (0.0) |
| Vaccine diphtheria, tetanus, pertussis, poliomyelitis and Haemophilus type B conjugates, adsorbed | 1 (0.1) | 5 (0.2) | 3 (0.0) | 0 (0.0) | 9 (0.0) |
| Cyanocobalamin | 1 (0.1) | 0 (0.0) | 2 (0.0) | 5 (0.0) | 8 (0.0) |
| Ipratropium bromide | 2 (0.1) | 2 (0.1) | 2 (0.0) | 2 (0.0) | 8 (0.0) |
| Potassium gluconate | 2 (0.1) | 4 (0.1) | 1 (0.0) | 1 (0.0) | 8 (0.0) |
| Dexamethasone | 3 (0.2) | 1 (0.0) | 1 (0.0) | 2 (0.0) | 7 (0.0) |
| Dexmedetomidine | 5 (0.3) | 0 (0.0) | 2 (0.0) | 0 (0.0) | 7 (0.0) |
| Flucytosine | 3 (0.2) | 1 (0.0) | 1 (0.0) | 2 (0.0) | 7 (0.0) |
| Hospital preparation of non-marketed medication | 0 (0.0) | 1 (0.0) | 1 (0.0) | 5 (0.0) | 7 (0.0) |
| Racecadotril | 0 (0.0) | 0 (0.0) | 0 (0.0) | 7 (0.1) | 7 (0.0) |
| Rocuronium bromide | 2 (0.1) | 3 (0.1) | 1 (0.0) | 1 (0.0) | 7 (0.0) |
| Lactulose | 3 (0.2) | 3 (0.1) | 0 (0.0) | 0 (0.0) | 6 (0.0) |
| Lansoprazole | 0 (0.0) | 0 (0.0) | 5 (0.1) | 1 (0.0) | 6 (0.0) |
| Remifentanil | 0 (0.0) | 0 (0.0) | 2 (0.0) | 4 (0.0) | 6 (0.0) |
| Sodium phenylbutyrate | 0 (0.0) | 0 (0.0) | 0 (0.0) | 6 (0.0) | 6 (0.0) |
| Tetanus immune globulin | 0 (0.0) | 1 (0.0) | 4 (0.0) | 1 (0.0) | 6 (0.0) |
| Tinzaparin sodium | 1 (0.1) | 0 (0.0) | 3 (0.0) | 2 (0.0) | 6 (0.0) |
| Tixocortol | 3 (0.2) | 1 (0.0) | 1 (0.0) | 1 (0.0) | 6 (0.0) |
| Treprostinil | 1 (0.1) | 0 (0.0) | 1 (0.0) | 4 (0.0) | 6 (0.0) |
| Beclometasone | 3 (0.2) | 1 (0.0) | 0 (0.0) | 1 (0.0) | 5 (0.0) |
| Isoprenaline | 0 (0.0) | 0 (0.0) | 4 (0.0) | 1 (0.0) | 5 (0.0) |
| Scopolamine | 0 (0.0) | 0 (0.0) | 2 (0.0) | 3 (0.0) | 5 (0.0) |
| Ticarcillin and clavulanate | 4 (0.2) | 1 (0.0) | 0 (0.0) | 0 (0.0) | 5 (0.0) |
| Tranexamic acid | 2 (0.1) | 0 (0.0) | 1 (0.0) | 2 (0.0) | 5 (0.0) |
| Ubidecarenone | 0 (0.0) | 1 (0.0) | 2 (0.0) | 2 (0.0) | 5 (0.0) |
| Budesonide and albuterol | 3 (0.2) | 1 (0.0) | 0 (0.0) | 0 (0.0) | 4 (0.0) |
| Carbamazepine | 0 (0.0) | 0 (0.0) | 1 (0.0) | 3 (0.0) | 4 (0.0) |
| Carbimazole | 1 (0.1) | 0 (0.0) | 1 (0.0) | 2 (0.0) | 4 (0.0) |
| Carglumic acid | 0 (0.0) | 0 (0.0) | 0 (0.0) | 4 (0.0) | 4 (0.0) |
| Magnesium sulfate | 0 (0.0) | 1 (0.0) | 0 (0.0) | 3 (0.0) | 4 (0.0) |
| Pancrealipase | 0 (0.0) | 0 (0.0) | 2 (0.0) | 2 (0.0) | 4 (0.0) |
| Tetracosactide | 3 (0.2) | 0 (0.0) | 0 (0.0) | 1 (0.0) | 4 (0.0) |
| Vigabatrin | 0 (0.0) | 0 (0.0) | 1 (0.0) | 3 (0.0) | 4 (0.0) |
| Bosentan | 0 (0.0) | 0 (0.0) | 2 (0.0) | 1 (0.0) | 3 (0.0) |
| Clarithromycin | 0 (0.0) | 0 (0.0) | 1 (0.0) | 2 (0.0) | 3 (0.0) |
| Mannitol | 0 (0.0) | 0 (0.0) | 0 (0.0) | 3 (0.0) | 3 (0.0) |
| Methylene blue | 2 (0.1) | 1 (0.0) | 0 (0.0) | 0 (0.0) | 3 (0.0) |
| Nadolol | 0 (0.0) | 0 (0.0) | 0 (0.0) | 3 (0.0) | 3 (0.0) |
| Neostigmine | 0 (0.0) | 1 (0.0) | 1 (0.0) | 1 (0.0) | 3 (0.0) |
| Octreotide | 0 (0.0) | 0 (0.0) | 2 (0.0) | 1 (0.0) | 3 (0.0) |
| Prazepam | 1 (0.1) | 0 (0.0) | 0 (0.0) | 2 (0.0) | 3 (0.0) |
| Acetazolamide | 0 (0.0) | 1 (0.0) | 1 (0.0) | 0 (0.0) | 2 (0.0) |
| Acetylcysteine | 0 (0.0) | 0 (0.0) | 1 (0.0) | 1 (0.0) | 2 (0.0) |
| Amphotericin B lipid complex | 0 (0.0) | 2 (0.1) | 0 (0.0) | 0 (0.0) | 2 (0.0) |
| Bethanechol | 0 (0.0) | 0 (0.0) | 1 (0.0) | 1 (0.0) | 2 (0.0) |
| Caspofungin | 1 (0.1) | 1 (0.0) | 0 (0.0) | 0 (0.0) | 2 (0.0) |
| Cefadroxil | 1 (0.1) | 1 (0.0) | 0 (0.0) | 0 (0.0) | 2 (0.0) |
| Cefoxitin | 1 (0.1) | 0 (0.0) | 1 (0.0) | 0 (0.0) | 2 (0.0) |
| Clopidrogel | 0 (0.0) | 0 (0.0) | 1 (0.0) | 1 (0.0) | 2 (0.0) |
| Clorazepate | 0 (0.0) | 0 (0.0) | 1 (0.0) | 1 (0.0) | 2 (0.0) |
| Dorzolamide | 1 (0.1) | 0 (0.0) | 0 (0.0) | 1 (0.0) | 2 (0.0) |
| Flecainide | 0 (0.0) | 0 (0.0) | 2 (0.0) | 0 (0.0) | 2 (0.0) |
| Human protein C | 0 (0.0) | 0 (0.0) | 1 (0.0) | 1 (0.0) | 2 (0.0) |
| Isoniazid | 0 (0.0) | 0 (0.0) | 0 (0.0) | 2 (0.0) | 2 (0.0) |
| Ketoconazole | 0 (0.0) | 1 (0.0) | 0 (0.0) | 1 (0.0) | 2 (0.0) |
| Latanoprost | 1 (0.1) | 0 (0.0) | 0 (0.0) | 1 (0.0) | 2 (0.0) |
| Melatonin | 0 (0.0) | 0 (0.0) | 0 (0.0) | 2 (0.0) | 2 (0.0) |
| Phloroglucinol | 0 (0.0) | 0 (0.0) | 0 (0.0) | 2 (0.0) | 2 (0.0) |
| Somatropin | 0 (0.0) | 0 (0.0) | 1 (0.0) | 1 (0.0) | 2 (0.0) |
| Spironolactone and trometamol | 1 (0.1) | 0 (0.0) | 1 (0.0) | 0 (0.0) | 2 (0.0) |
| Trolamine | 1 (0.1) | 0 (0.0) | 1 (0.0) | 0 (0.0) | 2 (0.0) |
| Vecuronium | 1 (0.1) | 0 (0.0) | 0 (0.0) | 1 (0.0) | 2 (0.0) |
| Chlorpromazine | 0 (0.0) | 0 (0.0) | 0 (0.0) | 1 (0.0) | 1 (0.0) |
| Desmopressin | 0 (0.0) | 0 (0.0) | 0 (0.0) | 1 (0.0) | 1 (0.0) |
| Desonide | 0 (0.0) | 0 (0.0) | 1 (0.0) | 0 (0.0) | 1 (0.0) |
| Fibrinogen concentrate (human) | 0 (0.0) | 0 (0.0) | 0 (0.0) | 1 (0.0) | 1 (0.0) |
| Hydroxychloroquine | 0 (0.0) | 0 (0.0) | 1 (0.0) | 0 (0.0) | 1 (0.0) |
| Hydroxycobalamin | 0 (0.0) | 0 (0.0) | 0 (0.0) | 1 (0.0) | 1 (0.0) |
| Iloprost | 0 (0.0) | 0 (0.0) | 1 (0.0) | 0 (0.0) | 1 (0.0) |
| Indomethacin | 0 (0.0) | 1 (0.0) | 0 (0.0) | 0 (0.0) | 1 (0.0) |
| Lopinavir and ritonavir | 0 (0.0) | 0 (0.0) | 1 (0.0) | 0 (0.0) | 1 (0.0) |
| Magnesium chloride | 0 (0.0) | 0 (0.0) | 1 (0.0) | 0 (0.0) | 1 (0.0) |
| Pamidronate | 0 (0.0) | 1 (0.0) | 0 (0.0) | 0 (0.0) | 1 (0.0) |
| Rotavirus vaccine | 0 (0.0) | 0 (0.0) | 0 (0.0) | 1 (0.0) | 1 (0.0) |
| Sodium hydroxybutyrate | 0 (0.0) | 0 (0.0) | 0 (0.0) | 1 (0.0) | 1 (0.0) |
| Timolol | 0 (0.0) | 0 (0.0) | 1 (0.0) | 0 (0.0) | 1 (0.0) |
| Trihexyphenidyl | 0 (0.0) | 0 (0.0) | 0 (0.0) | 1 (0.0) | 1 (0.0) |
| Warfarin | 0 (0.0) | 0 (0.0) | 0 (0.0) | 1 (0.0) | 1 (0.0) |

INN, International non-proprietary name
