# Supplementary material for: Characteristics of prescription in 29 Level 3 Neonatal Wards over a 2-year period (2017-2018). An inventory for future research
Source: PLoS One. 2019 Sep 19;14(9):e0222667. doi: 10.1371/journal.pone.0222667 (PMC6752821; doi:10.1371/journal.pone.0222667)
Supplement: S3 Table — (DOCX) [file pone.0222667.s003.docx]

**S3 Table**. Exposed neonates to the less prescribed medication International Non-Proprietary Names by route of administration in 29 French Level 3 Neonatal Wards (2017-2018)

|  | **Exposed neonates by route of administration** | | | | |
| --- | --- | --- | --- | --- | --- |
|  | **n=27382** | | | | |
|  | **Injectable** | **Oral** | **Respiratory** | **Ocular / Cutaneous** | **Others route** |
| **Medication INN prescription**, n (%) |  |  |  |  |  |
| Lactobacillus |  | 1457 (5.3) |  |  |  |
| Poractant alfa |  |  |  |  | 1417 (5.2) |
| Esomeprazole | 253 (0.9) | 1284 (4.7) |  |  |  |
| Sodium chloride | 797 (2.9) | 683 (2.5) |  | 7 (0.0) |  |
| Calcium folinate | 8 (0.0) | 1299 (4.7) |  |  |  |
| Glycerin |  |  |  |  | 1231 (4.5) |
| Fluconazole | 1195 (4.4) | 190 (0.7) |  |  |  |
| Hydrocortisone | 1049 (3.8) | 120 (0.4) |  | 1 (0.0) |  |
| Vaccine pneumococcal polysaccharide conjugates 13-valent absorbed | 1040 (3.8) |  |  |  |  |
| Metronidazole | 966 (3.5) | 158 (0.6) |  |  |  |
| Vaccine diphtheria, tetanus, acellular pertussis, hepatitis B, inactive poliomyelitis and haemophilus influenzae type B conjugate | 989 (3.6) |  |  |  |  |
| Insulin | 985 (3.6) |  |  |  |  |
| Dopamine | 934 (3.4) |  |  |  |  |
| Nystatin |  | 915 (3.3) |  |  |  |
| Norepinephrine tartrate | 912 (3.3) |  |  |  |  |
| Propofol | 909 (3.3) |  |  |  |  |
| Spironolactone |  | 883 (3.2) |  |  |  |
| Econazole |  |  |  | 857 (3.1) |  |
| Ibuprofen | 802 (2.9) |  |  |  |  |
| Sodium alginate and sodium bicarbonate |  | 776 (2.8) |  |  |  |
| Palivizumab | 742 (2.7) |  |  |  |  |
| Albumin | 740 (2.7) |  |  |  |  |
| Nalbuphine | 543 (2.0) |  |  |  | 139 (0.5) |
| Budesonide |  |  | 632 (2.3) |  |  |
| Lidocaine and prilocaine |  |  |  | 557 (2.0) |  |
| Picloxydine |  |  |  | 518 (1.9) |  |
| Phenobarbital | 505 (1.8) | 64 (0.2) |  |  |  |
| Tropicamide |  |  |  | 506 (1.8) |  |
| Fentanyl | 474 (1.7) |  |  | 8 (0.0) |  |
| Alfacalcidol |  | 448 (1.6) |  |  |  |
| Heparin | 402 (1.5) |  |  |  | 19 (0.1) |
| Calcium gluconate | 133 (0.5) | 291 (1.1) |  |  |  |
| Recombinant hepatitis B vaccine adsorbed | 407 (1.5) |  |  |  |  |
| Betamethasone | 271 (1.0) | 215 (0.8) |  |  |  |
| Phenylephrine |  |  |  | 402 (1.5) |  |
| Dobutamine | 394 (1.4) |  |  |  |  |
| Sodium bicarbonate | 379 (1.4) | 21 (0.1) |  |  |  |
| Epinephrine | 244 (0.9) |  | 127 (0.5) |  | 28 (0.1) |
| Pantoprazole | 385 (1.4) |  |  |  |  |
| Amphotericin B | 12 (0.0) | 341 (1.2) |  |  |  |
| Alprostadil | 305 (1.1) |  |  |  |  |
| Atracurium besilate | 292 (1.1) |  |  |  |  |
| Piperacillin and tazobactam | 289 (1.1) |  |  |  |  |
| Azithromycin |  | 73 (0.3) |  | 205 (0.7) |  |
| Mupirocin |  |  |  | 6 (0.0) | 262 (1.0) |
| Meropenem | 267 (1.0) |  |  |  |  |
| Cefepime | 262 (1.0) |  |  |  |  |
| Salbutamol | 30 (0.1) |  | 229 (0.8) |  |  |
| Ceftazidime | 242 (0.9) |  |  |  |  |
| Dimeticone |  | 240 (0.9) |  |  |  |
| Acyclovir | 231 (0.8) | 1 (0.0) |  | 49 (0.2) |  |
| Ranitidine | 158 (0.6) | 127 (0.5) |  |  |  |
| Nitric oxide |  |  |  |  | 205 (0.7) |
| Phosphore element |  | 202 (0.7) |  |  |  |
| Amoxicillin and clavulanate | 117 (0.4) | 118 (0.4) |  |  |  |
| Phenytoin | 188 (0.7) | 8 (0.0) |  |  |  |
| Levocarnitine | 156 (0.6) | 64 (0.2) |  |  |  |
| Cefazolin | 183 (0.7) |  |  |  |  |
| Ursodesoxycholic acid |  | 173 (0.6) |  |  |  |
| Zinc oxide |  |  |  | 171 (0.6) |  |
| Fluticasone |  |  | 169 (0.6) |  |  |
| Dexpanthenol |  |  |  | 162 (0.6) |  |
| Naloxone | 158 (0.6) |  |  |  | 3 (0.0) |
| Oxacillin | 161 (0.6) |  |  |  |  |
| Tuberculosis vaccine |  |  |  |  | 160 (0.6) |
| Doxapram | 149 (0.5) | 10 (0.0) |  |  |  |
| Josamycin |  | 146 (0.5) |  |  |  |
| Zidovudine | 51 (0.2) | 127 (0.5) |  |  |  |
| Piperacillin | 139 (0.5) |  |  |  |  |
| Retinol | 30 (0.1) |  |  | 107 (0.4) |  |
| Glucagon | 118 (0.4) |  |  |  | 18 (0.1) |
| Ornidazole | 123 (0.4) |  |  |  |  |
| Sorbitol |  |  |  |  | 123 (0.4) |
| Diazepam | 5 (0.0) | 103 (0.4) |  |  | 16 (0.1) |
| Ciclopirox |  |  |  | 120 (0.4) |  |
| Levetiracetam | 64 (0.2) | 93 (0.3) |  |  |  |
| Imipenem and cilastatin | 119 (0.4) |  |  |  |  |
| Ciprofloxacin | 110 (0.4) | 8 (0.0) |  | 1 (0.0) |  |
| Anti-hepatitis B immune globulin | 110 (0.4) |  |  |  |  |
| Filgrastim | 108 (0.4) |  |  |  |  |
| Hydroxyzine | 3 (0.0) | 95 (0.3) |  |  |  |
| Ceftriaxone | 96 (0.4) |  |  |  |  |
| Clonazepam | 74 (0.3) | 34 (0.1) |  |  |  |
| Folinic acid |  | 93 (0.3) |  |  |  |
| Micafungin | 92 (0.3) |  |  |  |  |
| Sildenafil | 1 (0.0) | 92 (0.3) |  |  |  |
| Immune glogulin | 91 (0.3) |  |  |  |  |
| Erythromycin | 3 (0.0) | 88 (0.3) |  |  |  |
| Levothyroxine sodium | 1 (0.0) | 90 (0.3) |  |  |  |
| Cefaclor |  | 89 (0.3) |  |  |  |
| Milrinone | 89 (0.3) |  |  |  |  |
| Linezolid | 33 (0.1) | 56 (0.2) |  |  |  |
| Vaccine diphtheria, tetanus, acellular pertussis, inactive poliomyelitis and haemophilus influenzae type B conjugate | 83 (0.3) |  |  |  |  |
| Omeprazole | 77 (0.3) | 6 (0.0) |  |  |  |
| Amiodarone | 2 (0.0) | 66 (0.2) |  |  |  |
| Glucose | 48 (0.2) | 19 (0.1) |  |  |  |
| Carbomer |  |  |  | 62 (0.2) |  |
| Bumetanide | 53 (0.2) |  |  |  |  |
| Benzylpenicillin | 52 (0.2) |  |  |  |  |
| Amphotericin B liposomal | 51 (0.2) |  |  |  |  |
| Clonidine | 42 (0.2) | 24 (0.1) |  |  |  |
| Tocofersolan | 13 (0.0) | 38 (0.1) |  |  |  |
| Adenosine phosphate | 50 (0.2) |  |  |  |  |
| Biotin | 13 (0.0) | 40 (0.1) |  |  |  |
| Methylprednisolone | 49 (0.2) |  |  |  |  |
| Lamivudine |  | 48 (0.2) |  |  |  |
| Pyridoxine | 40 (0.1) | 18 (0.1) |  |  |  |
| Rifampicin | 42 (0.2) | 9 (0.0) |  |  |  |
| Hydrochlorothiazide |  | 46 (0.2) |  |  |  |
| Propranolol | 10 (0.0) | 40 (0.1) |  |  |  |
| Fusidic acid |  |  |  | 44 (0.2) |  |
| Epoprostenol | 42 (0.2) |  |  |  |  |
| Nicardipine | 37 (0.1) | 12 (0.0) |  |  |  |
| Aluminium phosphate |  | 39 (0.1) |  |  |  |
| Nevirapine | 1 (0.0) | 39 (0.1) |  |  |  |
| Bifonazole |  |  |  | 37 (0.1) |  |
| Sodium polystyrene sulfonate |  | 10 (0.0) |  |  | 30 (0.1) |
| suxamethonium | 36 (0.1) |  |  |  |  |
| Valproic acid | 1 (0.0) | 35 (0.1) |  |  |  |
| Diosmectite |  | 34 (0.1) |  |  |  |
| Thiamine | 27 (0.1) | 16 (0.1) |  |  |  |
| Domperidone |  | 32 (0.1) |  |  |  |
| Teicoplanin | 32 (0.1) |  |  |  |  |
| Sulfamethoxazole and trimethoprim | 10 (0.0) | 23 (0.1) |  |  |  |
| Oxiconazole |  |  |  | 30 (0.1) |  |
| Trimebutine |  | 29 (0.1) |  | 1 (0.0) |  |
| Adenosine | 28 (0.1) |  |  |  |  |
| Lidocaine | 28 (0.1) |  |  |  |  |
| Clindamycin | 25 (0.1) | 1 (0.0) |  |  |  |
| Digoxin | 1 (0.0) | 25 (0.1) |  |  |  |
| Sodium benzoate | 25 (0.1) | 4 (0.0) |  |  |  |
| Cefixime |  | 25 (0.1) |  |  |  |
| Acebutolol |  | 24 (0.1) |  |  |  |
| Thiopental sodium | 23 (0.1) |  |  |  |  |
| Captopril |  | 22 (0.1) |  |  |  |
| Diazoxide |  | 22 (0.1) |  |  |  |
| Riboflavin |  | 22 (0.1) |  |  |  |
| Ganciclovir | 21 (0.1) |  |  |  |  |
| Oseltamivir |  | 21 (0.1) |  |  |  |
| Zinc |  | 21 (0.1) |  |  |  |
| Hyaluronic acid |  |  |  | 19 (0.1) |  |
| Silver nitrate |  |  |  | 18 (0.1) |  |
| Terbutaline |  |  | 18 (0.1) |  |  |
| Acetylsalicylic acid |  | 17 (0.1) |  |  |  |
| Carmellose |  |  |  | 17 (0.1) |  |
| Prednisolone |  | 17 (0.1) |  |  |  |
| Valganciclovir |  | 17 (0.1) |  |  |  |
| Cefamandole | 16 (0.1) |  |  |  |  |
| Alimemazine |  | 15 (0.1) |  |  |  |
| Desomedine |  |  |  | 15 (0.1) |  |
| Sulfadiazine |  | 11 (0.0) |  | 4 (0.0) |  |
| Theophylline | 13 (0.0) |  |  |  |  |
| Arginine | 5 (0.0) | 7 (0.0) |  |  |  |
| Flumazenil | 12 (0.0) |  |  |  |  |
| Miconazole |  | 12 (0.0) |  |  |  |
| Pyrimethamine |  | 12 (0.0) |  |  |  |
| Cethexonium bromide |  |  |  | 11 (0.0) |  |
| Norfloxacin |  |  |  | 11 (0.0) |  |
| Antithrombin III | 10 (0.0) |  |  |  |  |
| Fosfomycin | 10 (0.0) |  |  |  |  |
| Amitriptyline |  | 9 (0.0) |  |  |  |
| Fludrocortisone |  | 9 (0.0) |  |  |  |
| Ofloxacin |  |  |  | 8 (0.0) | 1 (0.0) |
| Phenoxymethyl penicillin |  | 9 (0.0) |  |  |  |
| Vaccine diphtheria, tetanus, pertussis, poliomyelitis and Haemophilus type B conjugates, adsorbed | 9 (0.0) |  |  |  |  |
| Cyanocobalamin |  | 6 (0.0) |  |  | 2 (0.0) |
| Ipratropium bromide |  |  | 8 (0.0) |  |  |
| Potassium gluconate |  | 8 (0.0) |  |  |  |
| Dexamethasone | 2 (0.0) |  |  | 5 (0.0) |  |
| Dexmedetomidine | 7 (0.0) |  |  |  |  |
| Flucytosine | 7 (0.0) | 1 (0.0) |  |  |  |
| Hospital preparation of non-marketed medication |  |  |  |  | 7 (0.0) |
| Racecadotril |  | 7 (0.0) |  |  |  |
| Rocuronium bromide | 7 (0.0) |  |  |  |  |
| Lactulose |  | 6 (0.0) |  |  |  |
| Lansoprazole |  | 6 (0.0) |  |  |  |
| Remifentanil | 6 (0.0) |  |  |  |  |
| Sodium phenylbutyrate |  | 6 (0.0) |  |  |  |
| Tetanus immune globulin | 6 (0.0) |  |  |  |  |
| Tinzaparin sodium |  |  |  |  | 6 (0.0) |
| Tixocortol |  |  |  |  | 6 (0.0) |
| Treprostinil | 6 (0.0) |  |  |  |  |
| Beclometasone |  |  | 5 (0.0) |  |  |
| Isoprenaline | 5 (0.0) |  |  |  |  |
| Scopolamine |  |  |  | 5 (0.0) |  |
| Ticarcillin and clavulanate | 5 (0.0) |  |  |  |  |
| Tranexamic acid | 5 (0.0) |  |  |  |  |
| Ubidecarenone |  | 5 (0.0) |  |  |  |
| Budesonide and albuterol |  |  | 4 (0.0) |  |  |
| Carbamazepine |  | 4 (0.0) |  |  |  |
| Carbimazole |  | 4 (0.0) |  |  |  |
| Carglumic acid |  | 4 (0.0) |  |  |  |
| Magnesium sulfate | 4 (0.0) |  |  |  |  |
| Pancrealipase |  | 4 (0.0) |  |  |  |
| Tetracosactide | 4 (0.0) |  |  |  |  |
| Vigabatrin |  | 4 (0.0) |  |  |  |
| Bosentan |  | 3 (0.0) |  |  |  |
| Clarithromycin |  | 3 (0.0) |  |  |  |
| Mannitol | 3 (0.0) |  |  |  |  |
| Methylene blue | 3 (0.0) |  |  |  |  |
| Nadolol |  | 3 (0.0) |  |  |  |
| Neostigmine | 2 (0.0) |  |  |  | 1 (0.0) |
| Octreotide | 3 (0.0) |  |  |  |  |
| Prazepam |  | 3 (0.0) |  |  |  |
| Acetazolamide | 1 (0.0) | 1 (0.0) |  |  |  |
| Acetylcysteine | 2 (0.0) |  |  |  |  |
| Amphotericin B lipid complex | 2 (0.0) |  |  |  |  |
| Bethanechol |  | 2 (0.0) |  |  |  |
| Caspofungin | 2 (0.0) |  |  |  |  |
| Cefadroxil |  | 2 (0.0) |  |  |  |
| Cefoxitin | 2 (0.0) |  |  |  |  |
| Clopidrogel |  | 2 (0.0) |  |  |  |
| Clorazepate |  | 2 (0.0) |  |  |  |
| Dorzolamide |  |  |  | 2 (0.0) |  |
| Flecainide |  | 2 (0.0) |  |  |  |
| Human protein C | 2 (0.0) |  |  |  |  |
| Isoniazid | 1 (0.0) | 1 (0.0) |  |  |  |
| Ketoconazole |  |  |  | 2 (0.0) |  |
| Latanoprost |  |  |  | 2 (0.0) |  |
| Melatonin |  | 2 (0.0) |  |  |  |
| Phloroglucinol | 2 (0.0) |  |  |  |  |
| Somatropin |  |  |  |  | 2 (0.0) |
| Spironolactone and trometamol | 2 (0.0) |  |  |  |  |
| Trolamine |  |  |  | 2 (0.0) |  |
| Vecuronium | 2 (0.0) |  |  |  |  |
| Chlorpromazine |  | 1 (0.0) |  |  |  |
| Desmopressin | 1 (0.0) |  |  |  |  |
| Desonide |  |  |  | 1 (0.0) |  |
| Fibrinogen concentrate (human) | 1 (0.0) |  |  |  |  |
| Hydroxychloroquine |  | 1 (0.0) |  |  |  |
| Hydroxycobalamin | 1 (0.0) |  |  |  |  |
| Iloprost |  |  | 1 (0.0) |  |  |
| Indomethacin |  | 1 (0.0) |  |  |  |
| Lopinavir and ritonavir |  | 1 (0.0) |  |  |  |
| Magnesium chloride |  | 1 (0.0) |  |  |  |
| Pamidronate | 1 (0.0) |  |  |  |  |
| Rotavirus vaccine |  | 1 (0.0) |  |  |  |
| Sodium hydroxybutyrate |  | 1 (0.0) |  |  |  |
| Timolol |  |  |  | 1 (0.0) |  |
| Trihexyphenidyl |  | 1 (0.0) |  |  |  |
| Warfarin |  | 1 (0.0) |  |  |  |

INN, International non-proprietary name
