# Supplementary material for: Characteristics of prescription in 29 Level 3 Neonatal Wards over a 2-year period (2017-2018). An inventory for future research
Source: PLoS One. 2019 Sep 19;14(9):e0222667. doi: 10.1371/journal.pone.0222667 (PMC6752821; doi:10.1371/journal.pone.0222667)
Supplement: S4 Table — (DOCX) [file pone.0222667.s004.docx]

**S4 Table**. Exposed neonates to the less prescribed medication International Non-Proprietary Names without any citation in Summary of Product Characteristics by gestational age in 29 French Level 3 Neonatal Wards (2017-2018)

|  | **Exposed neonates to medication INN without any citation in SmPC *** | | | | **Neonates exposed** |
| --- | --- | --- | --- | --- | --- |
|  | **by gestational age (weeks)** | | | | **medication INN** |
|  |  | | | | **without any** |
|  | **≤ 27** | **[28 - 31]** | **[32 - 36]** | **≥ 37** | **citation in SmPC *** |
|  | **n = 1740** | **n = 3293** | **n = 9350** | **n = 12999** | **n=27382** |
| **Medication INN prescription**, n (%) |  |  |  |  |  |
| phenobarbital | 48 (2.8) | 26 (0.8) | 76 (0.8) | 362 (2.8) | 512 (1.9) |
| fentanyl | 150 (8.6) | 80 (2.4) | 95 (1.0) | 157 (1.2) | 482 (1.8) |
| heparin | 85 (4.9) | 34 (1.0) | 69 (0.7) | 225 (1.7) | 413 (1.5) |
| betamethasone | 266 (15.3) | 42 (1.3) | 25 (0.3) | 70 (0.5) | 403 (1.5) |
| phenylephrine | 163 (9.4) | 169 (5.1) | 40 (0.4) | 30 (0.2) | 402 (1.5) |
| pantoprazole | 52 (3.0) | 38 (1.2) | 96 (1.0) | 199 (1.5) | 385 (1.4) |
| amphotericin B | 85 (4.9) | 105 (3.2) | 91 (1.0) | 71 (0.5) | 352 (1.3) |
| atracurium besilate | 113 (6.5) | 30 (0.9) | 35 (0.4) | 114 (0.9) | 292 (1.1) |
| piperacillin and tazobactam | 106 (6.1) | 45 (1.4) | 66 (0.7) | 72 (0.6) | 289 (1.1) |
| azithromycin | 53 (3.0) | 41 (1.2) | 88 (0.9) | 96 (0.7) | 278 (1.0) |
| meropenem | 139 (8.0) | 68 (2.1) | 35 (0.4) | 25 (0.2) | 267 (1.0) |
| cefepime | 117 (6.7) | 60 (1.8) | 35 (0.4) | 50 (0.4) | 262 (1.0) |
| salbutamol | 144 (8.3) | 30 (0.9) | 26 (0.3) | 50 (0.4) | 250 (0.9) |
| dimeticone | 10 (0.6) | 5 (0.2) | 98 (1.0) | 127 (1.0) | 240 (0.9) |
| ranitidine | 73 (4.2) | 63 (1.9) | 35 (0.4) | 56 (0.4) | 227 (0.8) |
| nitric oxide | 74 (4.3) | 28 (0.9) | 31 (0.3) | 72 (0.6) | 205 (0.7) |
| phenytoin | 9 (0.5) | 5 (0.2) | 29 (0.3) | 145 (1.1) | 188 (0.7) |
| levocarnitine | 37 (2.1) | 74 (2.2) | 42 (0.4) | 32 (0.2) | 185 (0.7) |
| cefazolin | 36 (2.1) | 9 (0.3) | 48 (0.5) | 90 (0.7) | 183 (0.7) |
| ursodesoxycholic acid | 66 (3.8) | 51 (1.5) | 30 (0.3) | 26 (0.2) | 173 (0.6) |
| zinc oxide | 20 (1.1) | 35 (1.1) | 84 (0.9) | 32 (0.2) | 171 (0.6) |
| fluticasone | 111 (6.4) | 37 (1.1) | 8 (0.1) | 13 (0.1) | 169 (0.6) |
| naloxone | 74 (4.3) | 17 (0.5) | 29 (0.3) | 41 (0.3) | 161 (0.6) |
| oxacillin | 75 (4.3) | 31 (0.9) | 17 (0.2) | 38 (0.3) | 161 (0.6) |
| doxapram | 113 (6.5) | 36 (1.1) | 1 (0.0) | 0 (0.0) | 150 (0.5) |
| epinephrine | 14 (0.8) | 8 (0.2) | 20 (0.2) | 106 (0.8) | 148 (0.5) |
| josamycin | 91 (5.2) | 33 (1.0) | 5 (0.1) | 17 (0.1) | 146 (0.5) |
| piperacillin | 69 (4.0) | 40 (1.2) | 15 (0.2) | 15 (0.1) | 139 (0.5) |
| glucagon | 2 (0.1) | 7 (0.2) | 56 (0.6) | 63 (0.5) | 128 (0.5) |
| sorbitol | 27 (1.6) | 50 (1.5) | 33 (0.4) | 13 (0.1) | 123 (0.4) |
| diazepam | 8 (0.5) | 6 (0.2) | 23 (0.2) | 85 (0.7) | 122 (0.4) |
| ciclopirox | 13 (0.7) | 26 (0.8) | 43 (0.5) | 38 (0.3) | 120 (0.4) |
| levetiracetam | 6 (0.3) | 7 (0.2) | 11 (0.1) | 96 (0.7) | 120 (0.4) |
| imipenem and cilastatin | 66 (3.8) | 23 (0.7) | 9 (0.1) | 21 (0.2) | 119 (0.4) |
| ciprofloxacin | 33 (1.9) | 27 (0.8) | 16 (0.2) | 39 (0.3) | 115 (0.4) |
| filgrastim | 50 (2.9) | 48 (1.5) | 6 (0.1) | 4 (0.0) | 108 (0.4) |
| hydroxyzine | 19 (1.1) | 8 (0.2) | 11 (0.1) | 59 (0.5) | 97 (0.4) |
| clonazepam | 7 (0.4) | 6 (0.2) | 12 (0.1) | 71 (0.5) | 96 (0.4) |
| folinic acid | 10 (0.6) | 32 (1.0) | 49 (0.5) | 2 (0.0) | 93 (0.3) |
| sildenafil | 18 (1.0) | 9 (0.3) | 11 (0.1) | 54 (0.4) | 92 (0.3) |
| cefaclor | 1 (0.1) | 3 (0.1) | 17 (0.2) | 68 (0.5) | 89 (0.3) |
| milrinone | 17 (1.0) | 7 (0.2) | 14 (0.1) | 51 (0.4) | 89 (0.3) |
| erythromycin | 30 (1.7) | 45 (1.4) | 10 (0.1) | 3 (0.0) | 88 (0.3) |
| linezolid | 55 (3.2) | 13 (0.4) | 7 (0.1) | 11 (0.1) | 86 (0.3) |
| omeprazole | 9 (0.5) | 5 (0.2) | 20 (0.2) | 47 (0.4) | 81 (0.3) |
| midazolam | 20 (1.1) | 5 (0.2) | 13 (0.1) | 41 (0.3) | 79 (0.3) |
| tropicamide | 20 (1.1) | 39 (1.2) | 12 (0.1) | 1 (0.0) | 72 (0.3) |
| amiodarone | 2 (0.1) | 4 (0.1) | 22 (0.2) | 39 (0.3) | 67 (0.2) |
| carbomer | 9 (0.5) | 3 (0.1) | 11 (0.1) | 39 (0.3) | 62 (0.2) |
| bumetanide | 23 (1.3) | 10 (0.3) | 7 (0.1) | 13 (0.1) | 53 (0.2) |
| amphotericin B liposomal | 41 (2.4) | 8 (0.2) | 1 (0.0) | 1 (0.0) | 51 (0.2) |
| clonidine | 25 (1.4) | 7 (0.2) | 7 (0.1) | 12 (0.1) | 51 (0.2) |
| adenosine phosphate | 1 (0.1) | 4 (0.1) | 18 (0.2) | 27 (0.2) | 50 (0.2) |
| biotin | 1 (0.1) | 2 (0.1) | 10 (0.1) | 36 (0.3) | 49 (0.2) |
| methylprednisolone | 7 (0.4) | 4 (0.1) | 9 (0.1) | 29 (0.2) | 49 (0.2) |
| lamivudine | 2 (0.1) | 3 (0.1) | 7 (0.1) | 36 (0.3) | 48 (0.2) |
| pyridoxine | 1 (0.1) | 2 (0.1) | 4 (0.0) | 41 (0.3) | 48 (0.2) |
| hydrochlorothiazide | 32 (1.8) | 13 (0.4) | 1 (0.0) | 0 (0.0) | 46 (0.2) |
| propranolol | 2 (0.1) | 4 (0.1) | 15 (0.2) | 25 (0.2) | 46 (0.2) |
| epoprostenol | 6 (0.3) | 7 (0.2) | 5 (0.1) | 24 (0.2) | 42 (0.2) |
| nicardipine | 8 (0.5) | 16 (0.5) | 5 (0.1) | 11 (0.1) | 40 (0.1) |
| aluminium phosphate | 1 (0.1) | 4 (0.1) | 21 (0.2) | 13 (0.1) | 39 (0.1) |
| nevirapine | 1 (0.1) | 2 (0.1) | 6 (0.1) | 30 (0.2) | 39 (0.1) |
| suxamethonium | 11 (0.6) | 4 (0.1) | 9 (0.1) | 12 (0.1) | 36 (0.1) |
| thiamine | 0 (0.0) | 3 (0.1) | 7 (0.1) | 24 (0.2) | 34 (0.1) |
| sulfamethoxazole and trimethoprim | 6 (0.3) | 3 (0.1) | 6 (0.1) | 16 (0.1) | 31 (0.1) |
| oxiconazole | 3 (0.2) | 10 (0.3) | 9 (0.1) | 8 (0.1) | 30 (0.1) |
| trimebutine | 5 (0.3) | 6 (0.2) | 6 (0.1) | 13 (0.1) | 30 (0.1) |
| adenosine | 1 (0.1) | 3 (0.1) | 5 (0.1) | 19 (0.1) | 28 (0.1) |
| lidocaine | 2 (0.1) | 3 (0.1) | 4 (0.0) | 19 (0.1) | 28 (0.1) |
| clindamycin | 14 (0.8) | 5 (0.2) | 3 (0.0) | 4 (0.0) | 26 (0.1) |
| sodium benzoate | 1 (0.1) | 1 (0.0) | 3 (0.0) | 21 (0.2) | 26 (0.1) |
| cefixime | 4 (0.2) | 1 (0.0) | 4 (0.0) | 16 (0.1) | 25 (0.1) |
| acebutolol | 2 (0.1) | 0 (0.0) | 10 (0.1) | 12 (0.1) | 24 (0.1) |
| captopril | 1 (0.1) | 2 (0.1) | 8 (0.1) | 11 (0.1) | 22 (0.1) |
| diazoxide | 0 (0.0) | 2 (0.1) | 4 (0.0) | 16 (0.1) | 22 (0.1) |
| riboflavin | 0 (0.0) | 3 (0.1) | 6 (0.1) | 13 (0.1) | 22 (0.1) |
| ganciclovir | 8 (0.5) | 3 (0.1) | 4 (0.0) | 6 (0.0) | 21 (0.1) |
| oseltamivir | 2 (0.1) | 5 (0.2) | 4 (0.0) | 10 (0.1) | 21 (0.1) |
| zinc | 10 (0.6) | 6 (0.2) | 4 (0.0) | 1 (0.0) | 21 (0.1) |
| glucose | 4 (0.2) | 3 (0.1) | 8 (0.1) | 4 (0.0) | 19 (0.1) |
| hyaluronic acid | 8 (0.5) | 6 (0.2) | 4 (0.0) | 1 (0.0) | 19 (0.1) |
| terbutaline | 6 (0.3) | 1 (0.0) | 3 (0.0) | 8 (0.1) | 18 (0.1) |
| acetylsalicylic acid | 0 (0.0) | 1 (0.0) | 3 (0.0) | 13 (0.1) | 17 (0.1) |
| carmellose | 2 (0.1) | 0 (0.0) | 1 (0.0) | 14 (0.1) | 17 (0.1) |
| prednisolone | 2 (0.1) | 1 (0.0) | 4 (0.0) | 10 (0.1) | 17 (0.1) |
| valganciclovir | 5 (0.3) | 3 (0.1) | 2 (0.0) | 7 (0.1) | 17 (0.1) |
| cefamandole | 5 (0.3) | 1 (0.0) | 6 (0.1) | 4 (0.0) | 16 (0.1) |
| alimemazine | 5 (0.3) | 1 (0.0) | 1 (0.0) | 8 (0.1) | 15 (0.1) |
| desomedine | 1 (0.1) | 3 (0.1) | 9 (0.1) | 2 (0.0) | 15 (0.1) |
| sulfadiazine | 3 (0.2) | 0 (0.0) | 2 (0.0) | 10 (0.1) | 15 (0.1) |
| theophylline | 7 (0.4) | 1 (0.0) | 2 (0.0) | 3 (0.0) | 13 (0.0) |
| miconazole | 0 (0.0) | 1 (0.0) | 3 (0.0) | 8 (0.1) | 12 (0.0) |
| pyrimethamine | 0 (0.0) | 0 (0.0) | 1 (0.0) | 11 (0.1) | 12 (0.0) |
| cethexonium bromide | 1 (0.1) | 2 (0.1) | 5 (0.1) | 3 (0.0) | 11 (0.0) |
| antithrombin III | 0 (0.0) | 1 (0.0) | 2 (0.0) | 7 (0.1) | 10 (0.0) |
| fosfomycin | 4 (0.2) | 4 (0.1) | 0 (0.0) | 2 (0.0) | 10 (0.0) |
| sodium polystyrene sulfonate | 1 (0.1) | 0 (0.0) | 3 (0.0) | 6 (0.0) | 10 (0.0) |
| amitriptyline | 0 (0.0) | 0 (0.0) | 1 (0.0) | 8 (0.1) | 9 (0.0) |
| ofloxacin | 0 (0.0) | 4 (0.1) | 3 (0.0) | 2 (0.0) | 9 (0.0) |
| cyanocobalamin | 1 (0.1) | 0 (0.0) | 2 (0.0) | 5 (0.0) | 8 (0.0) |
| ipratropium bromide | 2 (0.1) | 2 (0.1) | 2 (0.0) | 2 (0.0) | 8 (0.0) |
| potassium gluconate | 2 (0.1) | 4 (0.1) | 1 (0.0) | 1 (0.0) | 8 (0.0) |
| dexamethasone | 3 (0.2) | 1 (0.0) | 1 (0.0) | 2 (0.0) | 7 (0.0) |
| dexmedetomidine | 5 (0.3) | 0 (0.0) | 2 (0.0) | 0 (0.0) | 7 (0.0) |
| flucytosine | 3 (0.2) | 1 (0.0) | 1 (0.0) | 2 (0.0) | 7 (0.0) |
| hospital preparation of non-marketed medication | 0 (0.0) | 1 (0.0) | 1 (0.0) | 5 (0.0) | 7 (0.0) |
| racecadotril | 0 (0.0) | 0 (0.0) | 0 (0.0) | 7 (0.1) | 7 (0.0) |
| lactulose | 3 (0.2) | 3 (0.1) | 0 (0.0) | 0 (0.0) | 6 (0.0) |
| lansoprazole | 0 (0.0) | 0 (0.0) | 5 (0.1) | 1 (0.0) | 6 (0.0) |
| remifentanil | 0 (0.0) | 0 (0.0) | 2 (0.0) | 4 (0.0) | 6 (0.0) |
| tinzaparin sodium | 1 (0.1) | 0 (0.0) | 3 (0.0) | 2 (0.0) | 6 (0.0) |
| tixocortol | 3 (0.2) | 1 (0.0) | 1 (0.0) | 1 (0.0) | 6 (0.0) |
| treprostinil | 1 (0.1) | 0 (0.0) | 1 (0.0) | 4 (0.0) | 6 (0.0) |
| arginine | 0 (0.0) | 0 (0.0) | 1 (0.0) | 4 (0.0) | 5 (0.0) |
| beclometasone | 3 (0.2) | 1 (0.0) | 0 (0.0) | 1 (0.0) | 5 (0.0) |
| isoprenaline | 0 (0.0) | 0 (0.0) | 4 (0.0) | 1 (0.0) | 5 (0.0) |
| scopolamine | 0 (0.0) | 0 (0.0) | 2 (0.0) | 3 (0.0) | 5 (0.0) |
| ticarcillin and clavulanate | 4 (0.2) | 1 (0.0) | 0 (0.0) | 0 (0.0) | 5 (0.0) |
| tranexamic acid | 2 (0.1) | 0 (0.0) | 1 (0.0) | 2 (0.0) | 5 (0.0) |
| ubidecarenone | 0 (0.0) | 1 (0.0) | 2 (0.0) | 2 (0.0) | 5 (0.0) |
| budesonide and albuterol | 3 (0.2) | 1 (0.0) | 0 (0.0) | 0 (0.0) | 4 (0.0) |
| carbamazepine | 0 (0.0) | 0 (0.0) | 1 (0.0) | 3 (0.0) | 4 (0.0) |
| carbimazole | 1 (0.1) | 0 (0.0) | 1 (0.0) | 2 (0.0) | 4 (0.0) |
| carglumic acid | 0 (0.0) | 0 (0.0) | 0 (0.0) | 4 (0.0) | 4 (0.0) |
| magnesium sulfate | 0 (0.0) | 1 (0.0) | 0 (0.0) | 3 (0.0) | 4 (0.0) |
| tetracosactide | 3 (0.2) | 0 (0.0) | 0 (0.0) | 1 (0.0) | 4 (0.0) |
| vigabatrin | 0 (0.0) | 0 (0.0) | 1 (0.0) | 3 (0.0) | 4 (0.0) |
| bosentan | 0 (0.0) | 0 (0.0) | 2 (0.0) | 1 (0.0) | 3 (0.0) |
| clarithromycin | 0 (0.0) | 0 (0.0) | 1 (0.0) | 2 (0.0) | 3 (0.0) |
| gentamicin | 1 (0.1) | 1 (0.0) | 1 (0.0) | 0 (0.0) | 3 (0.0) |
| mannitol | 0 (0.0) | 0 (0.0) | 0 (0.0) | 3 (0.0) | 3 (0.0) |
| methylene blue | 2 (0.1) | 1 (0.0) | 0 (0.0) | 0 (0.0) | 3 (0.0) |
| nadolol | 0 (0.0) | 0 (0.0) | 0 (0.0) | 3 (0.0) | 3 (0.0) |
| neostigmine | 0 (0.0) | 1 (0.0) | 1 (0.0) | 1 (0.0) | 3 (0.0) |
| octreotide | 0 (0.0) | 0 (0.0) | 2 (0.0) | 1 (0.0) | 3 (0.0) |
| prazepam | 1 (0.1) | 0 (0.0) | 0 (0.0) | 2 (0.0) | 3 (0.0) |
| acetazolamide | 0 (0.0) | 1 (0.0) | 1 (0.0) | 0 (0.0) | 2 (0.0) |
| acetylcysteine | 0 (0.0) | 0 (0.0) | 1 (0.0) | 1 (0.0) | 2 (0.0) |
| amikacin | 2 (0.1) | 0 (0.0) | 0 (0.0) | 0 (0.0) | 2 (0.0) |
| amphotericin B lipid complex | 0 (0.0) | 2 (0.1) | 0 (0.0) | 0 (0.0) | 2 (0.0) |
| bethanechol | 0 (0.0) | 0 (0.0) | 1 (0.0) | 1 (0.0) | 2 (0.0) |
| caspofungin | 1 (0.1) | 1 (0.0) | 0 (0.0) | 0 (0.0) | 2 (0.0) |
| cefadroxil | 1 (0.1) | 1 (0.0) | 0 (0.0) | 0 (0.0) | 2 (0.0) |
| clopidrogel | 0 (0.0) | 0 (0.0) | 1 (0.0) | 1 (0.0) | 2 (0.0) |
| clorazepate | 0 (0.0) | 0 (0.0) | 1 (0.0) | 1 (0.0) | 2 (0.0) |
| dorzolamide | 1 (0.1) | 0 (0.0) | 0 (0.0) | 1 (0.0) | 2 (0.0) |
| flecainide | 0 (0.0) | 0 (0.0) | 2 (0.0) | 0 (0.0) | 2 (0.0) |
| isoniazid | 0 (0.0) | 0 (0.0) | 0 (0.0) | 2 (0.0) | 2 (0.0) |
| ketoconazole | 0 (0.0) | 1 (0.0) | 0 (0.0) | 1 (0.0) | 2 (0.0) |
| latanoprost | 1 (0.1) | 0 (0.0) | 0 (0.0) | 1 (0.0) | 2 (0.0) |
| melatonin | 0 (0.0) | 0 (0.0) | 0 (0.0) | 2 (0.0) | 2 (0.0) |
| phloroglucinol | 0 (0.0) | 0 (0.0) | 0 (0.0) | 2 (0.0) | 2 (0.0) |
| somatropin | 0 (0.0) | 0 (0.0) | 1 (0.0) | 1 (0.0) | 2 (0.0) |
| spironolactone and trometamol | 1 (0.1) | 0 (0.0) | 1 (0.0) | 0 (0.0) | 2 (0.0) |
| trolamine | 1 (0.1) | 0 (0.0) | 1 (0.0) | 0 (0.0) | 2 (0.0) |
| acyclovir | 0 (0.0) | 0 (0.0) | 1 (0.0) | 0 (0.0) | 1 (0.0) |
| chlorpromazine | 0 (0.0) | 0 (0.0) | 0 (0.0) | 1 (0.0) | 1 (0.0) |
| desonide | 0 (0.0) | 0 (0.0) | 1 (0.0) | 0 (0.0) | 1 (0.0) |
| hydroxychloroquine | 0 (0.0) | 0 (0.0) | 1 (0.0) | 0 (0.0) | 1 (0.0) |
| hydroxycobalamin | 0 (0.0) | 0 (0.0) | 0 (0.0) | 1 (0.0) | 1 (0.0) |
| iloprost | 0 (0.0) | 0 (0.0) | 1 (0.0) | 0 (0.0) | 1 (0.0) |
| indomethacin | 0 (0.0) | 1 (0.0) | 0 (0.0) | 0 (0.0) | 1 (0.0) |
| magnesium chloride | 0 (0.0) | 0 (0.0) | 1 (0.0) | 0 (0.0) | 1 (0.0) |
| pamidronate | 0 (0.0) | 1 (0.0) | 0 (0.0) | 0 (0.0) | 1 (0.0) |
| sodium hydroxybutyrate | 0 (0.0) | 0 (0.0) | 0 (0.0) | 1 (0.0) | 1 (0.0) |
| timolol | 0 (0.0) | 0 (0.0) | 1 (0.0) | 0 (0.0) | 1 (0.0) |
| trihexyphenidyl | 0 (0.0) | 0 (0.0) | 0 (0.0) | 1 (0.0) | 1 (0.0) |
| warfarin | 0 (0.0) | 0 (0.0) | 0 (0.0) | 1 (0.0) | 1 (0.0) |

INN, International non-proprietary name; SmPC, Summary of product characteristics.

** without any citation for the combination of the medication INN and its route of administration*
